# Local signal variability and functional connectivity: Sensitive measures of the excitation-inhibition ratio?

**DOI:** 10.1007/s11571-023-10003-x

**Published:** 2023-09-02

**Authors:** Anne M. van Nifterick, Elliz P. Scheijbeler, Alida A. Gouw, Willem de Haan, Cornelis J. Stam

**Affiliations:** 1 Alzheimer Center Amsterdam, Neurology, Vrije Universiteit Amsterdam, Amsterdam UMC Location VUmc, Amsterdam, The Netherlands; 2 Clinical Neurophysiology and MEG Center, Neurology, Vrije Universiteit Amsterdam, Amsterdam UMC Location VUmc, Amsterdam, The Netherlands; 3 Amsterdam Neuroscience, Neurodegeneration, Amsterdam, The Netherlands

**Keywords:** Joint permutation entropy, Signal variability, Functional connectivity, Network hyperexcitability, Alzheimer’s disease, Whole-brain computational modeling

## Abstract

A novel network version of permutation entropy, the inverted joint permutation entropy (JPE_inv_), holds potential as non-invasive biomarker of abnormal excitation-inhibition (E-I) ratio in Alzheimer’s disease (AD). In this computational modelling study, we test the hypotheses that this metric, and related measures of signal variability and functional connectivity, are sensitive to altered E-I ratios. The E-I ratio in each neural mass of a whole-brain computational network model was systematically varied. We evaluated whether JPE_inv_, local signal variability (by permutation entropy) and functional connectivity (by weighted symbolic mutual information (wsMI)) were related to E-I ratio, on whole-brain and regional level. The hub disruption index can identify regions primarily affected in terms of functional connectivity strength (or: degree) by the altered E-I ratios. Analyses were performed for a range of coupling strengths, filter and time-delay settings. On whole-brain level, higher E-I ratios were associated with higher functional connectivity (by JPE_inv_ and wsMI) and lower local signal variability. These relationships were nonlinear and depended on the coupling strength, filter and time-delay settings. On regional level, hub-like regions showed a selective decrease in functional degree (by JPE_inv_ and wsMI) upon a lower E-I ratio, and non-hub-like regions showed a selective increase in degree upon a higher E-I ratio. These results suggest that abnormal functional connectivity and signal variability, as previously reported in patients across the AD continuum, can inform us about altered E-I ratios.

**Supplementary Information:**

The online version contains supplementary material available at 10.1007/s11571-023-10003-x.

## Introduction

An increasing number of studies has provided evidence for an imbalance between excitatory and inhibitory (E-I) neuronal activity as key underlying mechanism of neurological disorders, including Alzheimer’s disease (AD), glioma (Numan et al. 2022), epilepsy (Houtman et al. 2021; Pani et al. 2021), and multiple sclerosis (Huiskamp et al. 2022). Especially because of the high failure rate of anti-amyloid clinical trials, there has been increased attention for other disease mechanisms of AD, such as abnormal E-I ratios, that may serve as therapeutic target to stabilize or prevent cognitive decline. AD patients seem to have altered synaptic activity and neuronal function as early as in a presymptomatic disease stage which may contribute to cognitive decline. For example, AD patients have an increased rate of subclinical epileptiform activity compared to controls and the presence of such events was associated with a faster progression in memory decline (Horvath et al. 2021; Vossel et al. 2016). Restoring the E-I ratio, therefore, provides a promising therapeutic (Bakker et al. 2015; Jin et al. 2022; Koch et al. 2022; Menardi et al. 2022; Vossel et al. 2021). Detection of (subclinical) epileptiform activity remains, however, challenging, especially in AD patients. Only a small percentage of AD patients experience seizures throughout the disease course and subclinical epileptiform activity occurs only sporadically. It is, furthermore, primarily reported during sleep and mostly in deeper layers of the cortex (Lam et al. 2017, 2020; Liedorp et al. 2010; Vossel et al. 2013). Besides the development of more accurate automatic spike detection algorithms (Furbass et al. 2020; Wilson and Emerson 2002), there is a need for alternative, quantitative, and more sensitive measures to timely capture abnormal E-I ratios in short noninvasive recordings.

Several neurophysiological measures have potential to identify abnormal E-I ratio. For instance, measures of cortical network hyperexcitability and epileptogenicity (indicating increased E-I ratios) include frequent small sharp spikes, temporal intermittent rhythmic delta activity and paroxysmal slow wave events (Yu et al. 2021). Interregional functional connectivity measures has been proposed as candidate marker of hyperexcitability in several studies (Cuesta et al. 2022; Ranasinghe et al. 2022a, b; Stam et al. 2023). Whereas band-pass power measures may be sensitive to total neuronal activity levels (Luppi et al. 2022; van Nifterick et al. 2022a, b), other neurophysiological measures, such as the steepness of the aperiodic component of the power spectrum (Donoghue et al. 2020; Gao et al. 2017) and the functional E-I (fEI) method (Bruining et al. 2020), serve as more direct indicators of E-I ratio. In a recent study, we proposed a novel network version of permutation entropy, the inverted joint permutation entropy (JPE_inv_), as a sensitive marker of E-I ratio. This measure integrates local signal variability and interregional connectivity, which both have been related to E-I in previous work (Bruining et al. 2020; Deco et al. 2014; Demirtas et al. 2017; Gao et al. 2017; Garrett et al. 2015; Maestu et al. 2021). The multi-scale character of JPE_inv_ offers a potential advantage in identifying disrupted E-I ratios from large-scale neurophysiological data when compared to methods constrained to a single scale (Scheijbeler et al. 2022). JPE_inv_ distinguished a small group of early-stage AD patients from healthy controls with a slightly higher accuracy than the relative theta power benchmark, which suggests its potentially added value as sensitive marker of E-I (Scheijbeler et al. 2022). However, whether and how JPE_inv_ is related to E-I remained to be investigated.

Investigating how E-I ratio changes translate to neurophysiological measures in human subjects is challenging, because it requires an experimental setup with E-I modulating therapeutics or invasive intracranial recordings. Computational models provide a means to manipulate local E-I ratios, according to pathophysiological mechanisms reported in the literature, and investigate its impact on large-scale neurophysiological activity. The biophysical meaning of neural mass model parameters in particular facilitate the integration of findings from experimental electrophysiology studies, conducted on the micro- and mesoscale, with human electroencephalography (EEG) or magnetoencephalography (MEG) studies, conducted on the macroscale. Furthermore, the realistic large-scale signals generated by these models can be subjected to analyses analogous to those applied to empirical neurophysiological data, and, thus, allowing for the application of the same analytical approaches employed in the prior JPE_inv_ study (Scheijbeler et al. 2022). Previous studies have already demonstrated that neurophysiological abnormalities associated with AD, such as spectral slowing and functional connectivity disruption, can be simulated by disrupting local neuronal activity in computational neural mass models(de Haan et al. 2012; Stefanovski et al. 2019; A. M. van Nifterick et al. 2022a, b). Virtual interventions that re-established E-I balance preserved and recovered the functional connectivity and network integrity (de Haan et al. 2017; Vattikonda et al. 2016). These studies show how computational models may help to better understand the mechanisms underlying abnormal neurophysiological activity in AD patients, and predict treatment effects. In addition, similar models can be used to implement local E-I ratio disruption associated with AD, and study its effect on JPE_inv_.

This study extends upon prior work with the aim to investigate whether JPE_inv_ is a potential surrogate measure of E-I. Through adapting the excitability of inhibitory interneuron populations in a computational neural mass network model, a potential underlying mechanism of abnormal E-I ratios in AD according to previous literature, we generated scenarios with varying E-I ratios. E-I ratios exist on multiple scales (*i.e*. synaptic, single neuron or circuit level), but in the current paper we refer to E-I ratios on a whole-brain network level (Sohal and Rubenstein 2019). To characterize to what extent JPE_inv_ and its association with E-I ratio are determined by the underlying symbolic dynamics based measures of local signal variability and functional connectivity between regions, we also performed permutation entropy (PE) and weighted symbolic mutual information (wsMI) analyses. We investigated the relationships between these neurophysiological measures with E-I ratio on whole-brain and regional level. We also computed the hub disruption index (for JPE_inv_ and wsMI) to study whether regions with a high intrinsic level functional connectivity (so-called hubs) were specifically vulnerable to E-I ratio changes. This multi-scale approach will significantly improve our knowledge on how E-I ratio changes translate to local dynamics, interregional functional connectivity, and functional brain network organization.

We here propose a method that could help to identify E-I ratio changes in a non-invasive manner using short term neurophysiological recordings. This could, ultimately, facilitate selection of neurological patients eligible for future clinical trials that target the E-I ratio.

## Materials and methods

### The computational brain network model

**Fig. 1:**
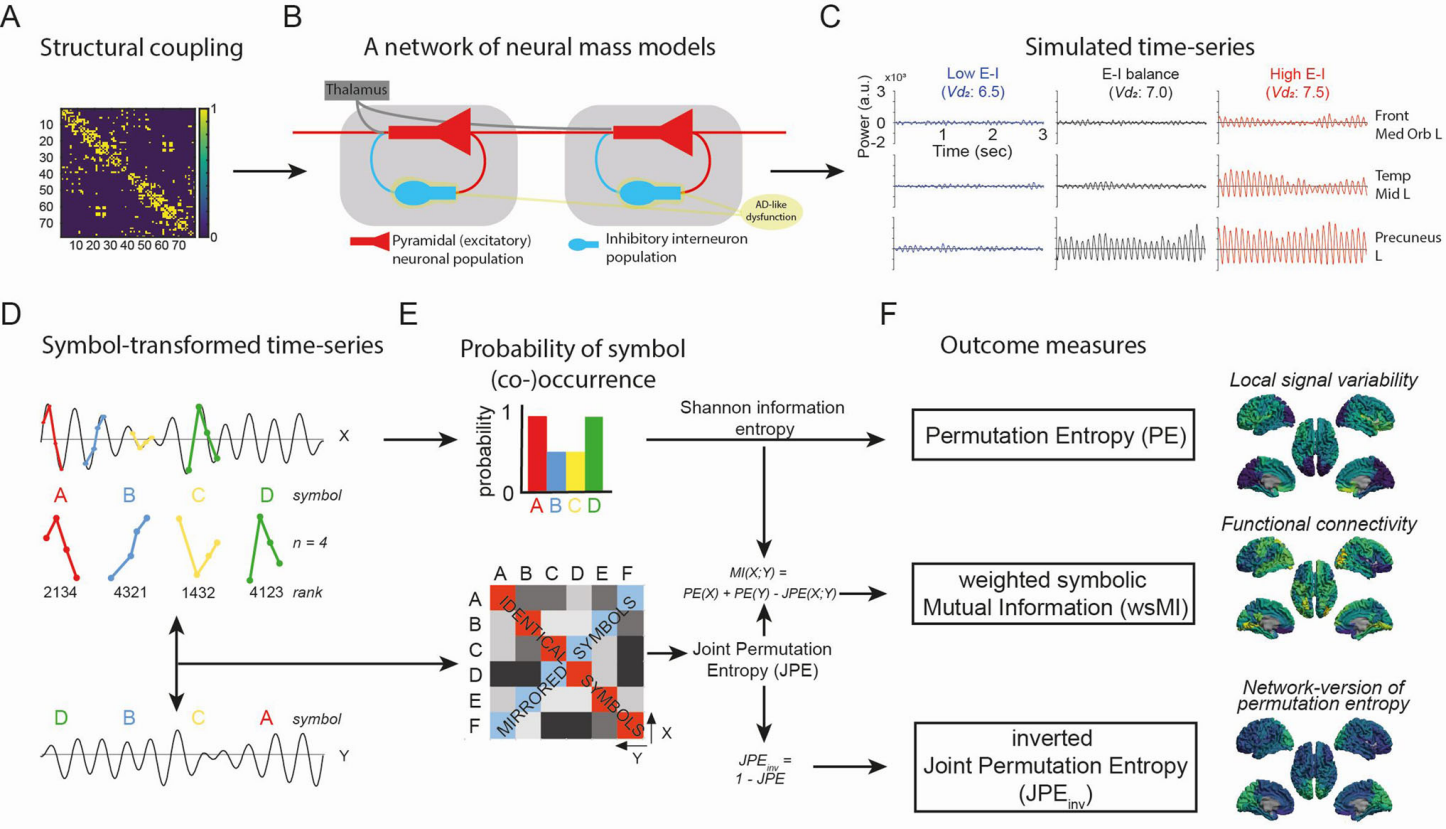
Flow chart of simulated EEG data generation and analyses. **a** 78 neural mass models were coupled according to a healthy human structural connectivity matrix (Gong et al. 2009). **b** Each neural mass consisted of an excitatory pyramidal neuronal population (in red) and inhibitory interneuron population (in blue). The firing threshold potential of the inhibitory interneuron population (*Vd*_*2*_) of each neural mass was adjusted to simulate AD pathology and obtain a network with altered excitation-inhibition (E-I) ratios. **c** Example traces of simulated cortical activity in three cortical regions in models of varying E-I ratio. **d** Each simulated time series was transformed into a sequence of symbols. This was accomplished by an ordinal ranking of the amplitudes of each time point in a vector of length *n*. **e**–**f** The variability in the probability distribution of the symbol occurrence per cortical region was evaluated by permutation entropy (PE). The probability of the co-occurrence of symbols between two regions X and Y was calculated, and a volume conduction correction was applied. The variability in co-occurrence probability was measured by the joint permutation entropy (JPE) which was then used to estimate the weighted symbolic mutual information (wsMI). The JPE was inverted to enable an intuitive interpretation, where higher JPE_inv_ indicates stronger connectivity. Vd_2_: Inhibitory interneuron excitability threshold potential; Front Med Orb L: Frontal Medial Orbital cortex, left hemisphere; Temp Mid L: Temporal medial cortex, left hemisphere; *n* = number of consecutive data points in the vector; JPE_inv_: inverted joint permutation entropy

The computational brain network model used in this study consisted of 78 coupled neural masses. The neural mass was originally developed to explain the human resting-state cortical alpha oscillations and involved an excitatory neuronal population and an inhibitory interneuron population (Lopes da Silva et al. 1974). The excitatory and inhibitory neuronal populations were coupled to each other. Each neural mass was considered one of the 78 cortical regions of the Automated Anatomical Labeling (AAL) atlas (Supplementary Table 1) (Gong et al. 2009; Tzourio-Mazoyer et al. 2002). Neural masses were coupled according to an average human diffusion tensor imaging based structural connectivity matrix (Gong et al. 2009) (Fig. 1a, b). Coupling between neural masses was always reciprocal and excitatory. A number of parameters (Table 1) described the average activity of the excitatory and inhibitory neuronal population. Each neuronal population had a membrane potential parameter, determined by *V*_*e*_*(t)* for excitatory and *V*_*i*_*(t)* for inhibitory populations. The membrane potentials were influenced by impulse responses, which reflected the postsynaptic activity. The impulse responses were taken from (Zetterberg et al. 1978) and can be described as follows:

**Table 1:** Computational neural mass model parameters, descriptions and (range of) values

Parameter	Description	Value	Range, steps
*t*	Sample time	0.002 s	
*P(t)*	The pulse density of an input signal to the excitatory population	480 spikes/s^−1^	
*Noise*	Random fluctuations around average level of P(t)	1.0	
*A h*_*e*_*(t)*	Amplitude of the EPSP	1.6 mV	
*A h*_*i*_*(t)*	Amplitude of the IPSP	32 mV	
*a h*_*e*_*(t)*	Shape parameter of EPSP	55 s^−1^	
*b h*_*e*_*(t)*	Shape parameter of EPSP	605 s^−1^	
*a h*_*i*_*(t)*	Shape parameter of IPSP	27.5 s^−1^	
*b h*_*i*_*(t)*	Shape parameter of IPSP	55 s^−1^	
*g*	Parameter of sigmoid function that relates membrane potential to impulse density	25 s^−1^	
*q*	Parameter of sigmoid function that relates membrane potential to impulse density	0.34 mV^−1^	
*Vd*_*1*_	Threshold potential used in the sigmoid function that relates membrane potential to impulse density for main population of excitatory neurons	7 mV	
*Vd*_*2*_	Threshold potential used in sigmoid function that relates membrane potential to impulse density for inhibitory neurons	7 mV	6–8, 0.5
*C1*	Connection strength between main population of excitatory and inhibitory neurons	32	
*C2*	Connection strength between inhibitory neurons and main population of excitatory neurons	3	
*S*	Coupling strength between neural masses (gain factor)	1.0	0–2, 0.5
*T*	Time delay for the coupling between neural masses	0.002 s	
*N*	Number of neural masses (/nodes) in network model	78	

$$h\left(\tau \right)=\mathrm{A}\left[\mathrm{exp}\left(-a\tau \right)-\mathrm{exp}\left(-\mathrm{b\tau }\right)\right]\,\mathrm{for} \,\tau \ge 0$$

$$h\left(\tau \right)=0\,\mathrm{for}\,\tau <0$$

Upon an impulse response, the change in membrane potential was transferred into pulse density (or spike density) by the sigmoid functions S1(x) and S2(x), which were also adapted from Zetterberg et al*.* (1978):

$$S\left[Vm-Vd\right]=g\mathrm{exp}\left\{q\left(Vm-Vd\right)\right\}\,\mathrm{for}\,Vm\le Vd$$

$$S\left[Vm-Vd\right]=g\left[2-\mathrm{exp}\left\{q\left(Vd-Vm\right)\right\}\right]\mathrm{ for} Vm>Vd$$

Within each neural mass, the excitatory to inhibitory coupling within each neural mass was described by *C1* and the inhibitory to excitatory coupling by *C2*. Coupled neural masses received excitatory input from other neural masses with a fixed delay (*T*) and of strength (*S*). Each neural mass received additional excitatory input from a thalamic region, with a random fluctuating pulse density *P(t).*

In this study, we altered the *Vd*_*2*_ parameter, which served as a firing threshold potential for inhibitory interneurons. Higher values of *Vd*_*2*_ indicate that the inhibitory neuronal populations need more excitatory input in order to pulse. Inhibitory interneurons activity is important for the generation of local oscillatory activity and functional connectivity at the large-scale brain level (Deco et al. 2014). The excitability of inhibitory interneurons were specifically altered because of their importance in (early) AD (Martinez-Losa et al. 2018; Palop & Mucke 2016; Scaduto et al. 2023; Verret et al. 2012). Disrupted inhibitory interneuron function has previously been related to increased firing rates of pyramidal neurons and oscillatory slowing, the most robust neurophysiological signature of human AD patients (Engels et al. 2017; Gouw et al. 2017; Luppi et al. 2022), in computational whole-brain network models (Stefanovski et al. 2019; van Nifterick et al. 2022a, b). We, therefore, homogeneously and systematically varied inhibitory interneuron excitability parameter (*Vd*_*2*_) for each neural mass model in the network to alter whole-brain E-I ratio associated with AD (Palop & Mucke 2016; van Nifterick et al. 2022a, b), within a range of 6 to 8 (mV) and with steps of 0.5. E-I ratios were calculated by the excitatory and inhibitory firing rates (E/(E + I)) and averaged across all cortical regions. To confirm that altered excitability of inhibitory interneurons within neural masses indeed changed whole-brain E-I ratio, we plotted the E-I ratio (averaged across all cortical regions) as function of *Vd*_*2*_.

The main output of a neural mass model is the fluctuation of the average membrane potential of the excitatory neuronal population across time (Fig. 1c). These fluctuations simulate the neurophysiological resting-state alpha activity as measured by EEG or MEG and can, thus, also be analyzed as such. Other model output includes the pulse density of the inhibitory and excitatory neuronal populations per unit time, which can be interpreted as a measure of neuronal population firing rate. This is of value as it provides a way to quantify E-I ratios.

Settings of the model parameters are provided in Table 1 and were based on previous studies (Lopes da Silva et al. 1974; Ponten et al. 2010; Stam et al. 1999; Ursino et al. 2007; Zetterberg et al. 1978). The whole-brain coupling parameter (*S*) determines the strength of the interactions between coupled neural mass models. Previous studies commonly used *S*-values between 0.5 and 1.5 to create networks that generate dynamics characteristic of being near a phase transition (de Haan et al. 2012; Ponten et al. 2010; Tewarie et al. 2019; van Dellen et al. 2013; A. M. van Nifterick et al. 2022a, b). This is important because the cortex is thought to operate near a critical point, balanced between chaos and order, or between asynchrony and synchrony, which is needed for optimal information processing (Beggs 2008, 2019, 2022; O'Byrne and Jerbi 2022; Xu et al. 2022). Here, *S* was not restricted to one value but varied between 0 and 2.0, with steps of 0.5. This allows to study how the relationships between E-I ratio and the neurophysiological outcome measures depend on coupling strength.

We ran each model (with a different *Vd*_*2*_) 10 times, using a sample time of 0.002 s, resulting in 40960 samples (or 81.92 s). The model is available from and analyses were performed using an in-house developed software (Brainwave, version *0.9.163.26*, available from home.kpn.nl /stam7883/brainwave.html).

#### Local signal variability

Signal variability of the simulated oscillations per cortical region was quantified by permutation entropy (PE). PE is a robust measure and captures the predictability or regularity of a signal (Fig. 1d–f), see (Bandt and Pompe 2002). Neurophysiological signals that are highly variable, complex, not repetitive, and not predictable have high entropy (close to 1). Neurophysiological signal that are less variable, more simple and repetitive will have low entropy (close to 0). Details about how PE is calculated per region are provided in the Supplementary Material.

#### Interregional functional connectivity

Information sharing between two brain regions was quantified by the weighted symbolic mutual information (wsMI). The wsMI estimates the extent to which two brain regions present non-random co-occurring symbols (Fig. 1d–f), as explained previously by (Yin et al. 2019). Further details about the mathematical computation of the wsMI can be found in the Supplementary Material. In this modeling study, signal spread and volume conduction are not an issue, but, to allow comparison to previous studies, we performed a similar volume conduction correction as by (King et al. 2013; Scheijbeler et al. 2022). Two brain regions that are functionally connected will have a higher wsMI than regions with a weak or no functional connection.

#### Network-version of permutation entropy

The JPE_inv_ is a multivariate version of permutation entropy and is a nonlinear coupling measure. JPE measures the variability in the co-occurrence of symbols (Fig. 1d–f). The JPE was inverted (hence JPE_inv_) to facilitate an intuitive interpretation as a measure of functional connectivity, as was suggested previously (Scheijbeler et al. 2022). Higher functional connectivity between two regions will therefore lead to higher JPE_inv_ values (closer to 1). Further details on JPE_inv_ computation are provided in the Supplementary Material.

#### The symbolic dynamics parameter settings

Two parameters need to be set for symbolic dynamics analyses: the embedding dimension (*n)* and the time-delay (*τ*). We set *n* to 4 such that the length of *n!* (24) is much smaller than the length of the investigated time series (4096 samples per iteration), as was recommended previously (Bandt and Pompe 2002). The results could therefore also be compared to the findings of Scheijbeler et al. (2022). Time-delay *τ* reflects the number of time points between the samples included in the computation of the symbolic dynamics metrics (i.e. PE, wsMI and JPE_inv_). It therefore determines the time scale under investigation. Previous studies showed the importance of the time-scale to the magnitude and direction of differences found in symbolic dynamics analysis (Costa et al. 2002; Goldberger et al. 2002). Selecting the appropriate time scale for analysis depends on the research question and data type (Kosciessa et al. 2020). The computational model used in this study primarily generates alpha like oscillations. Simulated broadband data will therefore not show full correspondence to physiological broadband data. Previous modeling work did however show that a reduction in inhibitory interneuron excitability could generate slower (theta-like) oscillatory activity (van Nifterick et al. 2022a, b). To analyze both fast and slow events, we first present findings for *τ* = 1 for broadband (0.5–70 Hz). We also present findings for *τ* = 1 for (extended) alpha band (6–13 Hz), to capture dynamics on slower time scales. We expected that, since the simulated broadband power spectrum is dominated by power in the alpha band, the results for broadband and extended alpha band would be similar. As alternative to narrow-band pass filtering, broadband data was also analyzed with a higher *τ*-value. We explored the effect of a range of *τ*-values on the relationship between JPE_inv_ and E-I ratio (Supplementary Fig. 1). For *τ*-values > 16 the relationship was independent of *τ* (Supplementary Fig. 1). We also analyzed broadband data with *τ* = 50 to capture dynamics on slow time scales.

#### Whole-brain statistical analyses

To obtain whole-brain average results, PE, wsMI and JPE_inv_ results were averaged across runs (*n* = 10) and regions (*n* = 78). The first part of whole-brain level statistical analyses were performed for a subset of models that are believed to best represent the (patho)physiological nature of brain oscillations close to a phase transition: a model with E-I balance (*Vd*_*2*_: 7.0, *S*: 1.0), a model with low E-I ratio (*Vd*_*2*_: 6.5, *S*: 1.0) and a model with high E-I ratio (*Vd*_*2*_: 7.5, *S*: 1.0). We performed independent *t*-tests to compare PE, wsMI and JPE_inv_ between the model with E-I balance and both high and low E-I ratio models, respectively. Cohen’s *d* was computed to measure effect sizes. The second part of the whole-brain level statistical analyses were performed for a larger subset of models, that span the full range of E-I ratios (*Vd*_*2*_ range: 6.0–8.0, with steps of 0.5) with a single coupling (*S*-) value of 1.0. A Pearson’s *r* correlation coefficient analysis was performed to measure the association between the inhibitory interneuron excitability parameter *Vd*_*2*_ and PE, wsMI and JPE_inv,_ respectively. A Pearson’s *r* correlation coefficient analysis was also performed to directly measure the association between the E-I ratio and PE, wsMI and JPE_inv,_ respectively. In addition, a Pearson’s *r* correlation coefficient analyses was performed to study the association between JPE_inv_ and PE and between JPE_inv_ and wsMI. A *p*-value < 0.05 was considered statistically significant.

#### Regional statistical analyses and the hub disruption index

For regional analysis, we selected a similar subset of models close to a phase transition: a model with E-I balance (*Vd*_*2*_: 7.0, *S*: 1.0), a model with a relatively low E-I ratio (*Vd*_*2*_: 6.5, *S*: 1.0) and a model with a relatively high E-I ratio (*Vd*_*2*_: 7.5, *S*: 1.0). We first computed the connectivity by both wsMI and JPE_inv_ between each pair of regions (averaged across 10 runs), resulting in a 78 × 78 matrix for each connectivity measure. We then calculated the wsMI- and JPE_inv_- based functional connectivity strength (or: functional degree) per region, that is, the average functional connectivity of that region compared to all other regions. Regional differences in PE and functional degree based on wsMI and JPE_inv_ between high or low E-I models and the model with E-I balance were tested using multiple non-parametric Mann–Whitney U tests: a comparison of ranks. A False Discovery Rate (FDR) approach was applied using the two-stage linear step-up procedure of Benjamini, Krieger and Yekutielie and using a desired FDR of 1%. To summarize the functional network reorganization after E-I ratio perturbation, the hub disruption index (HDI) was computed (Achard et al. 2012; Termenon et al. 2016). The difference in functional degree of a region in a high or low E-I ratio model compared to the functional degree of that region in the model with E-I balance was plotted as function of the regional degree in the model with E-I balance. A simple linear regression model was fit to the data and the slope defined the HDI. A significantly negative HDI can indicate that the impact of E-I ratio changes is dependent on the initial level of functional degree. Depending on the offset, it indicates that regions with a higher degree in models with balanced E-I ratios are primarily disrupted by altered E-I ratios, or that regions with a lower degree in models with balanced E-I ratios primarily show an increase in degree by altered E-I ratios, or it can be a combination of these two.

All statistical analyses were performed in GraphPad Prism version 9.3.1 for Windows, GraphPad Software, San Diego, California USA, www.graphpad.com.

## Results

### Inhibitory interneuron excitability alters E-I ratio

**Fig. 2:**
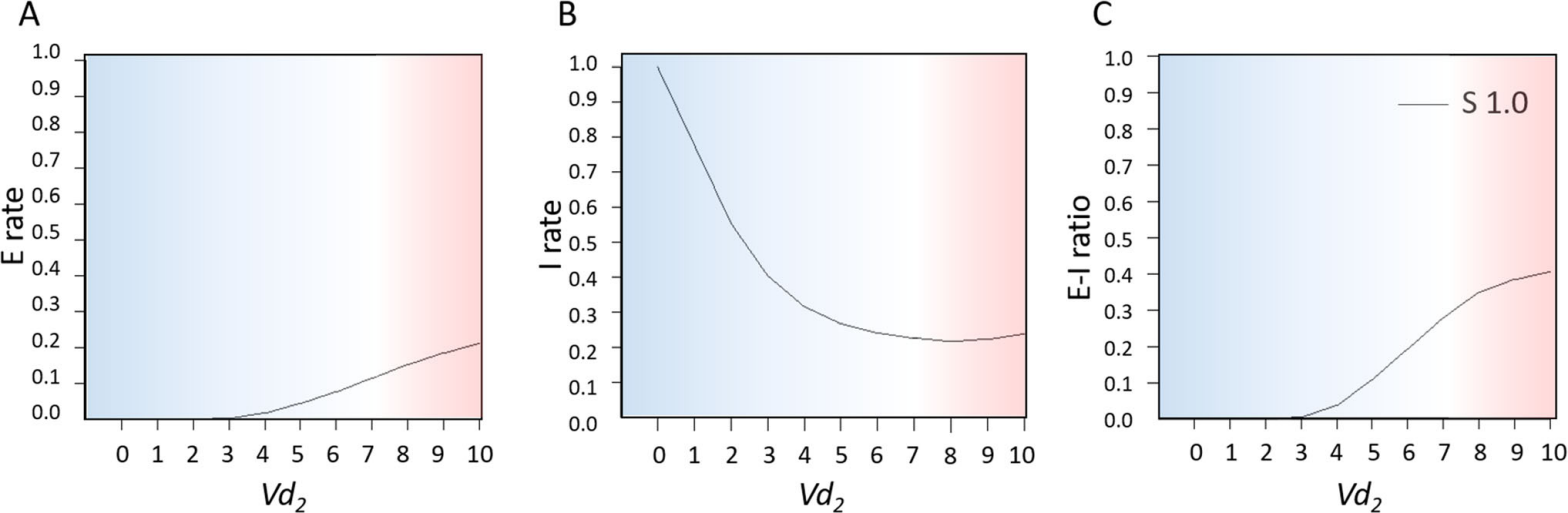
A higher inhibitory interneuron firing threshold potential (*Vd*_*2*_) is associated with higher E-I ratios. In a model with E-I balance, the inhibitory interneuron excitability threshold parameter (*Vd*_*2*_) has a value of 7 (depicted  on a white background). **a**–**c** A higher *Vd*_*2*_ (depicted on a red background) is associated with a whole-brain average increase in excitatory neuronal population firing activity (E rate) (**a**), a reduction of inhibitory interneuron population firing rate (I rate) (**b**), and an increase in the whole-brain average E-I ratio (**c**). A lower *Vd*_*2*_ resulted in opposite effects, presented in blue. Rates were normalized between 0 and 1, and E-I ratio = E/(E + I). *S*, coupling strength

To change the model E-I ratio, we altered the *Vd*_*2*_ model parameter homogeneously across the 78 cortical regions. The *Vd*_*2*_ model parameter determined the inhibitory interneurons excitability. Perturbation of the inhibitory interneuron excitability indeed generated changes in firing rates of excitatory and inhibitory neuronal populations and disrupted the E-I ratio on whole-brain level (Fig. 2). Lower inhibitory interneuron excitability (by a higher *Vd*_*2*_) resulted in higher excitatory neuronal firing rates (Fig. 2a), lower inhibitory firing rates (Fig. 2b), and higher E-I ratios (Fig. 2c). It was, therefore, considered an appropriate parameter to simulate AD-related abnormal E-I ratio.

### Whole-brain level PE, wsMI and JPE_inv_ relate to E-I ratio

On whole-brain level, all three outcome measures (PE, wsMI and JPE_inv_) showed a nonlinear relationship with E-I ratio (determined by the *Vd*_*2*_ parameter) and coupling strength (*S*). Statistical comparisons of whole-brain PE, wsMI and JPE_inv_ between a subset of high (*Vd*_*2*_: 6.5, S: 1.0) or low E-I ratio (*Vd*_*2*_: 7.5, S1.0) models and a model with E-I balance (*Vd*_*2*_7.0, *S*: 1.0) confirmed significant differences (Supplementary Table 2). These findings indicate that JPE_inv_, but also PE and wsMI are markers of E-I. Cohen’s *d* effect sizes were large for all measures (Supplementary Table 2). The JPE_inv_ showed a larger mean effect size compared to signal variability and functional connectivity by wsMI for 6–13 Hz data specifically. These results suggest that a small change in E-I ratio can cause a large difference in signal variability and functional connectivity measured by both wsMI and JPE_inv_.

**Fig. 3:**
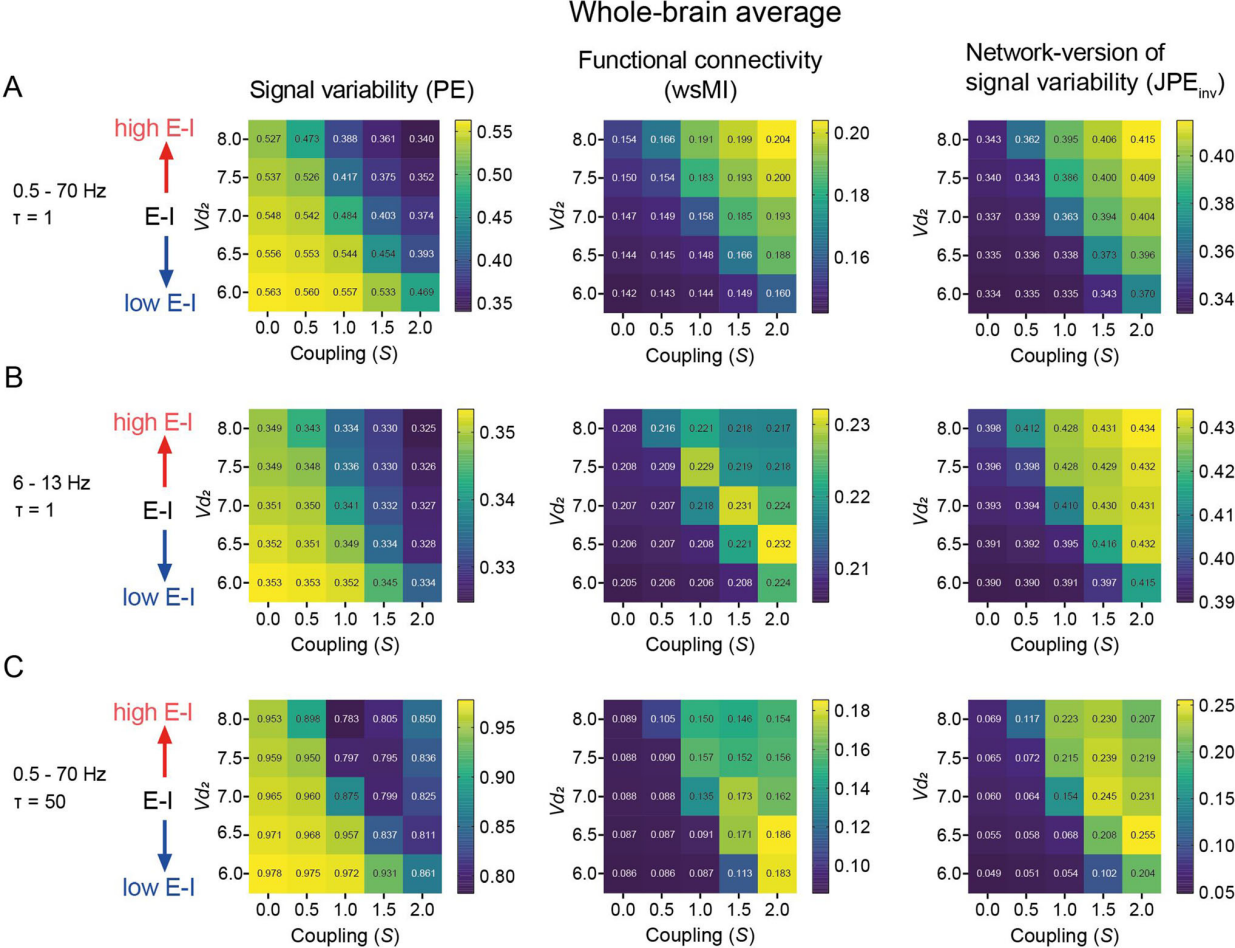
Whole-brain level PE, wsMI and JPE_inv_ relate to E-I ratio and coupling strength. **a**–**c** Whole-brain average local signal variability was measured by permutation entropy (PE), and functional connectivity by weighted symbolic mutual information (wsMI) and inverted joint permutation entropy (JPE_inv_). Results presented for varying levels of E-I ratios, determined by the inhibitory interneuron firing threshold potential (*Vd*_*2*_), and coupling strength (*S*). Each cell in a *heatmap* plot presents an average value calculated from data across all 78 regions and over 10 runs. Analyses were repeated for different time scales (by time-delay *τ*, or narrow band-pass filtering). Yellow/brighter colors mean higher values and blue/darker colors mean lower values

Signal variability was lower (PE values closer to 0) in models with higher E-I ratios (Fig. 3a–c, *left column*). While the relationship between E-I ratio and signal variability remained consistent, the magnitude of whole brain signal variability and its variation across models with varying E-I ratio differed when examined across various time scales (Fig. 3a–c, *left column*). Correlation analyses showed a robust correlation between signal variability and *Vd*_*2*_, the control parameter of E-I ratio, across time scales (Supplementary Fig. 2, Supplementary Table 3). Signal variability at whole-brain level was highest for *τ* = 50 (characterizing slow dynamics), followed by *τ* = 1 for broadband (capturing mixed dynamics on fast and slow time scales), and for 6–13 Hz (characterizing dynamics in the alpha band specifically) (Fig. 3a–c, *left column*).

The relationship between functional connectivity (measured by both wsMI and JPE_inv_) and E-I ratio was in the opposite direction of the relationship between signal variability and E-I ratio: models with higher E-I ratios showed higher connectivity than models with lower E-I ratios (Fig. 3a–c, *middle and right column*). The magnitude of functional connectivity at whole-brain depended on the time scale under analyses. Functional connectivity by wsMI and JPE_inv_ was highest for 6–13 Hz, followed by *τ* = 50 and *τ* = 1 for broadband. Correlation analyses revealed a significant association between the E-I ratio control parameter *Vd*_*2*_ and JPE_inv_ for all investigated time scales (Supplementary Fig. 2, Supplementary Table 3). More diverse patterns appeared for the correlation analyses between E-I and functional connectivity by wsMI across time scales. A significant correlation was found for *τ* = 50, and *τ* = 1 for broadband, but not for 6–13 Hz (Supplementary Fig. 2, Supplementary Table 3). This result is probably explained by the inversed U-shape relationship between E-I ratio and functional connectivity by wsMI, which is most evident for 6–13 Hz (Fig. 3b, *middle column*). Mainly for this time scale, but also for *τ* = 50, functional connectivity by wsMI was maximal for models that just crossed the transition point (Fig. 3b–c, *middle column*), at a somewhat high E-I ratio. Maximal functional connectivity by JPE_inv_ could also be observed at a similar (somewhat high) E-I ratio (Fig. 3c, r*ight column*), but only for *τ* = 50 and strong coupling values (*S* > 1.0). After this point, further increasing the E-I ratio does not further increase the functional connectivity on whole-brain level, but can actually lead to a reduction of connectivity. These findings indicate that the relationship between JPE_inv_ and E-I ratio is more robust than wsMI because it is less influenced by time scale. An additional correlation analyses between the actual E-I ratio (instead of *Vd*_*2*_) and the outcome measures signal variability (PE), functional connectivity by wsMI and JPE_inv_ showed similar results (Supplementary Fig. 3, Supplementary Table 4). These findings support the hypothesis that JPE_inv,_ but also signal variability and functional connectivity by wsMI, are markers of E-I.

Correlation analyses also showed that a higher JPE_inv_ was significantly associated with higher wsMI (Supplementary Fig. 4, Supplementary Table 5) and lower signal variability (Supplementary Fig. 4, Supplementary Table 5). These significant associations were present for all time scales (Supplementary Fig. 4, Supplementary Table 5). These results suggest that JPE_inv_ indeed simultaneously measures both signal variability and functional connectivity. Despite these significant associations, correlation analyses showed a stronger association between JPE_inv_ and signal variability than between JPE_inv_ and wsMI. This would mean that, although JPE_inv_ includes wsMI, JPE_inv_ may also provide information not captured by wsMI under certain conditions.

### Region-dependent relationships between PE, wsMI, JPE_inv_ and E-I ratio

Furthermore, we investigated whether the relationship between the E-I ratio and both signal variability and functional connectivity (as measured by wsMI and JPE_inv_) depended on the region of interest.

**Fig. 4:**
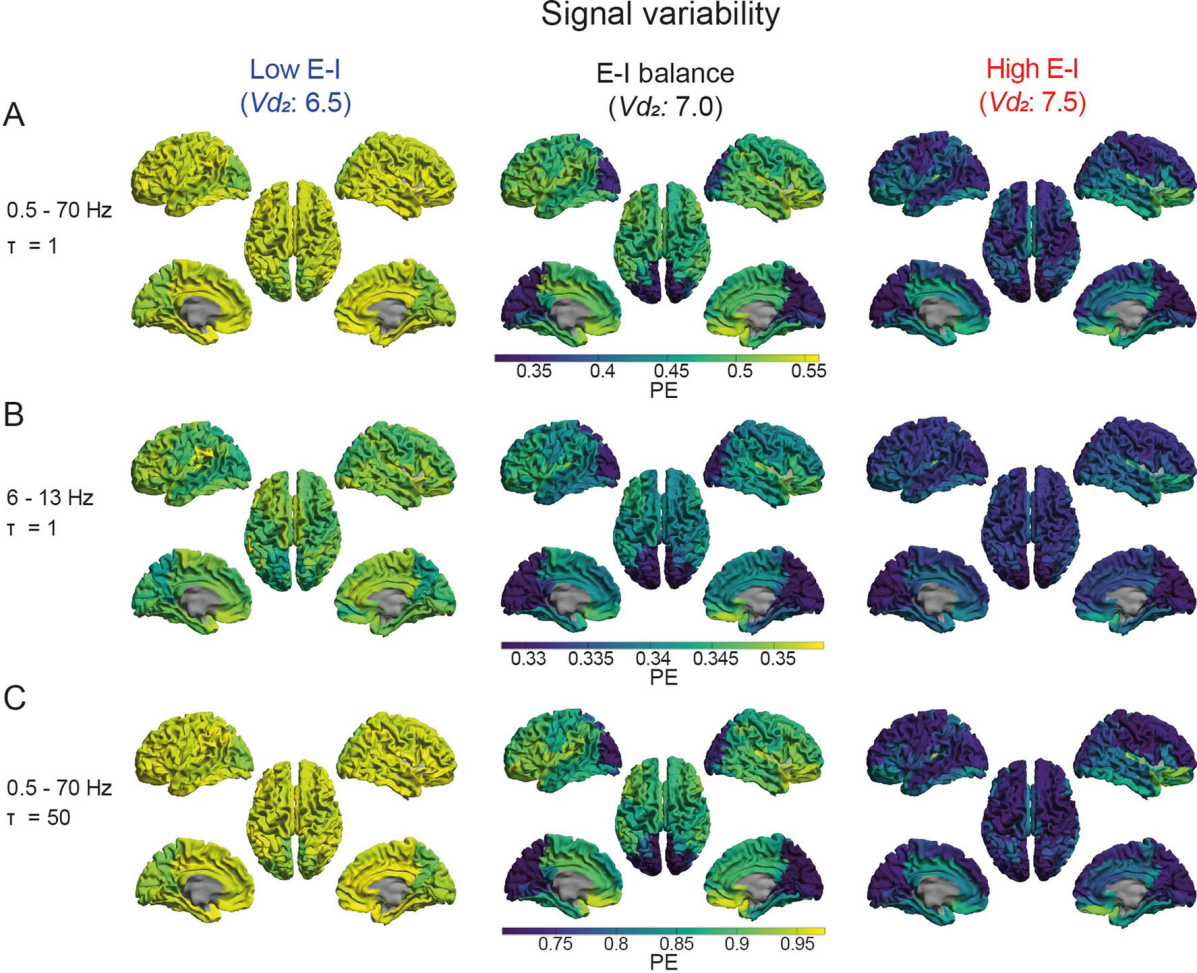
A region-dependent relationship between signal variability and E-I ratio. **a**–**c** The *brain color* plots show the regional level of signal variability (PE). Lower values are depicted in blue/dark colors and higher values in yellow/bright colors. Results are presented for model with varying levels of E-I, as determined by the inhibitory interneuron firing threshold parameter (*Vd*_*2*_). Analyses were repeated for different time scales. PE: permutation entropy*; τ*: time-delay between samples

In the selected decreased E-I ratio model (*Vd*_*2*_: 6.5, *S*: 1.0), signal variability seemed predominantly *increased* in regions with *lower* signal variability in the model with E-I balance (*Vd*_*2*_: 7.0, *S*: 1.0), such as the occipital regions (Fig. 4, Supplementary Fig. 5). In the selected increased E-I ratio model (*Vd*_*2*_: 7.5, *S*: 1.0) signal variability seemed predominantly *decreased* in regions with *higher* signal variability in the model with E-I balance (*Vd*_*2*_: 7.0, *S*: 1.0), such as the frontal, central and parietal regions (Fig. 4, Supplementary Fig. 5). These findings seemed to be largely independent of the time scale under analysis. Together, these results suggest a region-dependent relationship between signal variability and E-I ratio.

**Fig. 5:**
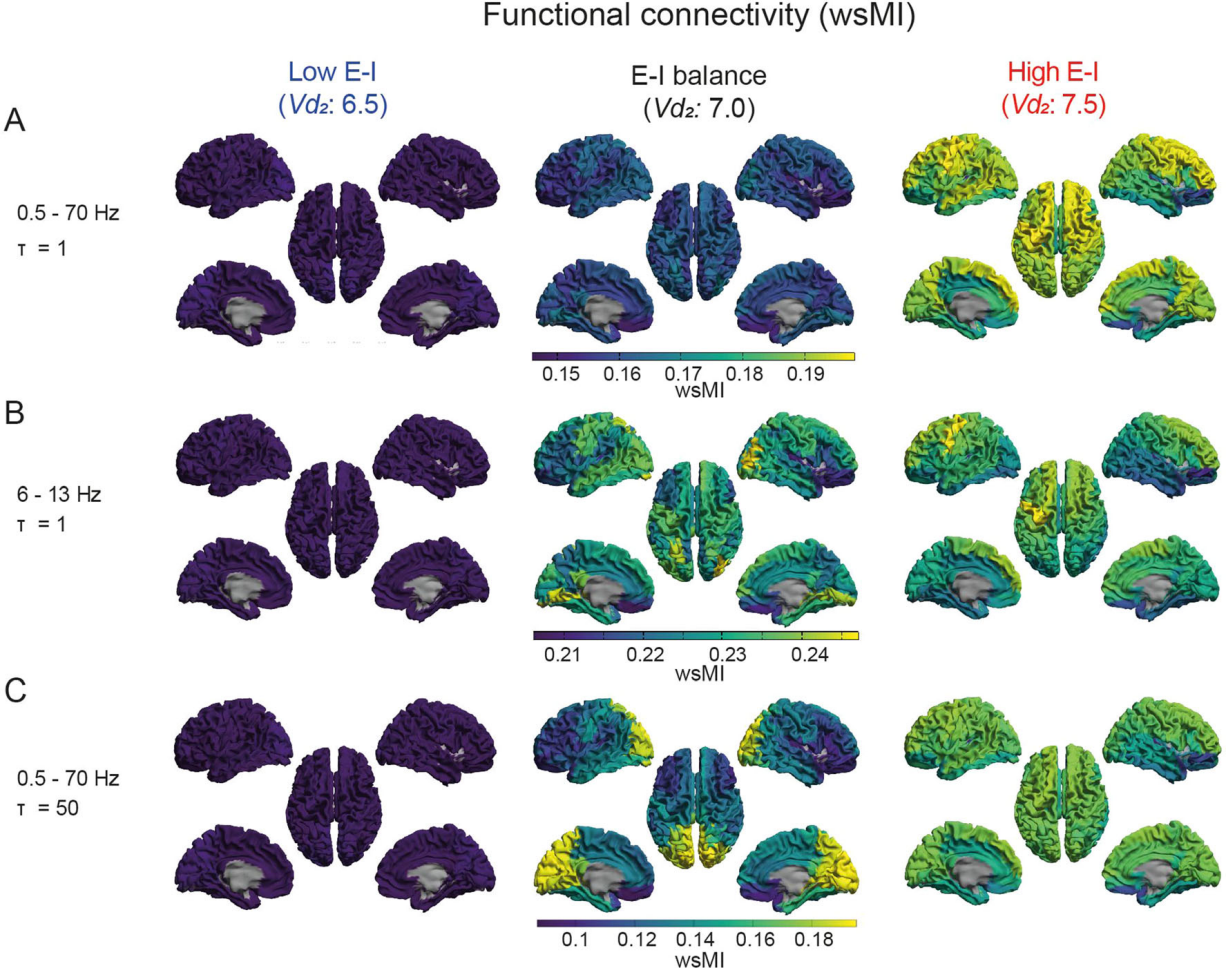
A region-dependent relationship between wsMI and E-I ratio. **a**–**c** The *brain color* plots present weighted symbolic mutual information (wsMI) per region, obtained by averaging the connectivity between that region and all other regions. The results are presented for models with varying levels of E-I, as determined by the inhibitory interneuron firing threshold potential (*Vd*_*2*_). Lower connectivity levels are depicted in blue/dark colors and higher values in yellow/brighter colors. Analyses were repeated for different time scales. wsMI: weighted symbolic mutual information; *τ*: time-delay between samples

**Fig. 6:**
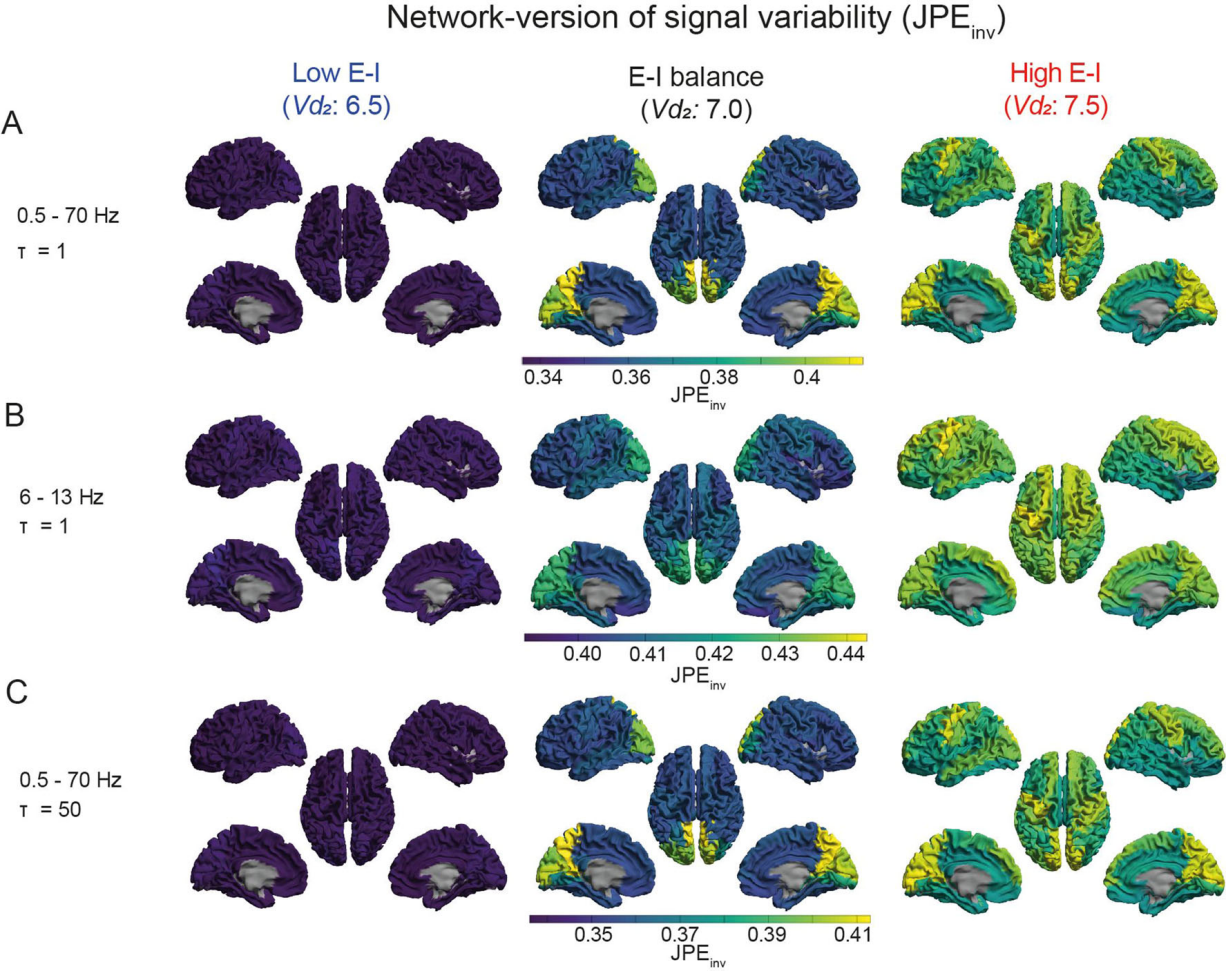
A region-dependent relationship between JPE_inv_ and E-I ratio. **a**–**c** The *brain color* plots present inverted joint permutation entropy (JPE_inv_) per region, obtained by averaging the connectivity between that region and all other regions. The results are presented for models with varying levels of E-I, as determined by the inhibitory interneuron firing threshold potential (*Vd*_*2*_). Lower connectivity levels are depicted in blue/dark colors and higher values in yellow/brighter colors. Analyses were repeated for different time scales (**a**–**c**). JPE_inv_: inverted joint permutation entropy; *τ*: time-delay between samples

**Fig. 7:**
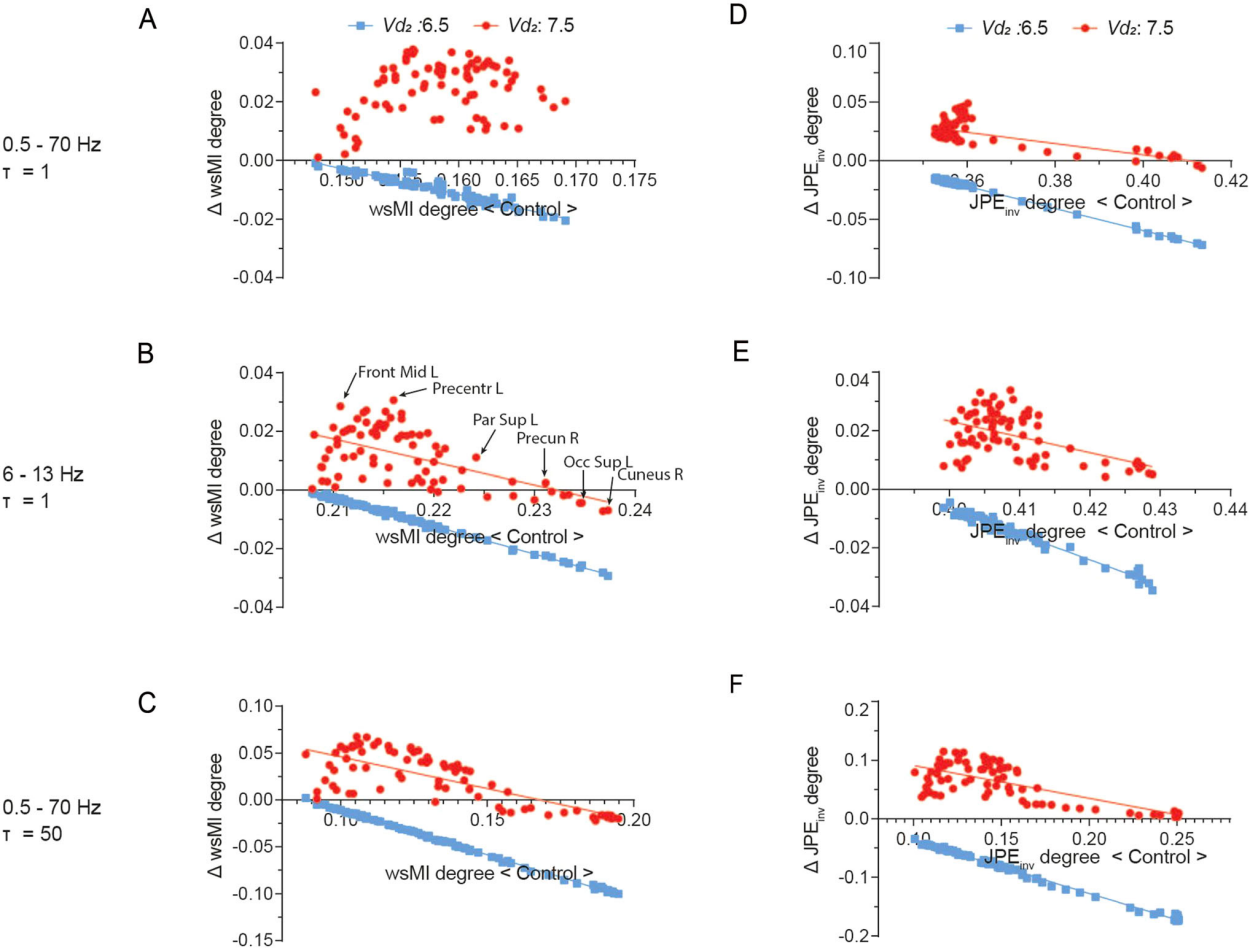
A degree-dependent impact of abnormal E-I ratio on functional connectivity. **a**–**c** The *y*-axis of the *scatter* plots show the regional change in functional degree by wsMI and JPE_inv_ after E-I ratio disruption (low E-I ratio (*Vd*_*2*_: 6.5) in blue and high E-I ratio (*Vd*_*2*_: 7.5) in red), compared to the functional degree in that region in a model with E-I balance (*Vd*_*2*_: 7.0). This difference is plotted as function of the regional degree in the model with E-I balance, on the *x*-axis. Analyses were repeated for different time scales. The (significant) linear regression models with the best fit are shown as solid lines

Functional connectivity by wsMI was generally increased across the cortex upon an increase in E-I ratio, in particular in regions that showed lower functional connectivity in the model with E-I balance, such as frontal and central regions (Fig. 5, Supplementary Fig. 5–6). For regions with a very high level of functional connectivity by wsMI in the model with E-I balance in 6 -13 Hz and *τ* = 50 specifically, however, an increase in E-I ratio resulted in a decrease in functional connectivity (Fig. 5, Supplementary Fig. 5b, c, *middle column*). Upon a decrease in E-I ratio, the functional connectivity measure was decreased across the cortex, particularly in regions with a high functional connectivity in the model with E-I balance, such as the parieto-occipital and temporal regions (Fig. 5, Supplementary Fig. 5–6). Similar effects were found for functional connectivity by JPE_inv_ (Fig. 6, Supplementary Fig. 5, Supplementary Fig. 7). These findings were further quantified by the hub disruption index (HDI) (Fig. 7, Table 2). The HDI analysis confirmed a selective disruption of hub-like regions (*i.e.* regions with a high average functional connectivity level, or degree), in terms of changes in their functional connectivity to other regions, upon a decreased E-I ratio (Fig. 7). We also found a selective increase of average functional degree in regions with initially low and intermediate degree (or: non-hubs), upon an increased E-I ratio (Fig. 7b–f). The region-specific sensitivity of functional connectivity by wsMI and JPE_inv_ to E-I ratio perturbation was relatively independent of the time scale under analysis. These analyses revealed a specific, degree-dependent disruption of functional connectivity by wsMI and JPE_inv_ upon an altered E-I ratio.

**Table 2:** The hub disruption index revealed a selective, degree-dependent disruption of functional connectivity upon altered E-I ratio. The results of simple linear regression analyses showed a significant association between the functional degree in a model with E-I balance (as reference model) and the change in functional degree by both wsMI and JPE_inv_ upon altered E-I ratios

	Offset		Slope		*R*^*2*^		*DFn, DFd*		*F*-statistic		*p*-value	
	Low E-I *Vd*_*2*_: 6.5	High E-I *Vd*_*2*_: 7.5	Low E-I *Vd*_*2*_: 6.5	High E-I *Vd*_*2*_: 7.5	Low E-I *Vd*_*2*_: 6.5	High E-I *Vd*_*2*_: 7.5	Low E-I *Vd*_*2*_: 6.5	High E-I *Vd*_*2*_: 7.5	Low E-I *Vd*_*2*_: 6.5	High E-I *Vd*_*2*_: 7.5	Low E-I *Vd*_*2*_: 6.5	High E-I *Vd*_*2*_: 7.5
*0.5–70 Hz* *τ = 1*												
wsMI	0.130	0.110	− 0.885	0.500	.934	.069	1, 76	1, 76	1069	5.655	< .001	.020
JPE_inv_	0.316	0.198	− 0.938	− 0.482	.997	.469	1, 76	1, 76	24,615	67.11	< .001	< .001
*6–13 Hz* *τ = 1*												
wsMI	0.198	0.183	− 0.957	− 0.787	.995	.344	1, 76	1, 76	16,569	39.79	< .001	< .001
JPE_inv_	0.347	0.239	− 0.883	− 0.540	.973	.276	1, 76	1, 76	2758	28.94	< .001	< .001
*0.5–70 Hz* *τ = 50*												
wsMI	0.084	0.114	− 0.951	− 0.677	.998	.588	1, 76	1, 76	46,155	108.5	< .001	< .001
JPE_inv_	0.052	0.147	− 0.903	− 0.561	.995	.526	1, 76	1, 76	13,806	84.23	< .001	< .001

## Discussion

This computational modeling study showed that a network version of permutation entropy, JPE_inv_, is sensitive to changes in E-I ratio, and may therefore serve as surrogate marker for E-I. Local signal variability (PE) and functional connectivity by wsMI also related to E-I ratio. On whole-brain level, the relationship between signal variability, functional connectivity (by JPE_inv_ or wsMI) and E-I ratio was dependent on the coupling strength, and the filter- and time-delay settings. JPE_inv_ represented both signal variability and functional connectivity by wsMI, with the former exhibiting a stronger relationship. Whole-brain level JPE_inv_ showed the largest change upon alterations in E-I ratio for alpha oscillations, which suggests its additive value in determining E-I ratios compared to the single-scale measures signal variability and functional connectivity by wsMI. On regional level, the ‘hubness’ of a region determined the susceptibility of wsMI- and JPE_inv_-based functional connectivity disruption to abnormal E-I ratios.

**Fig. 8:**
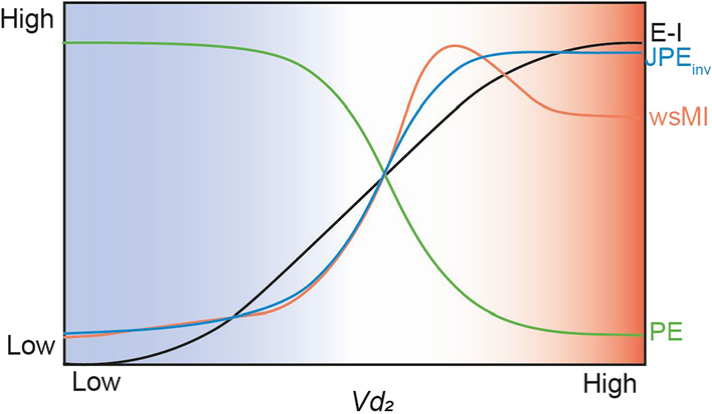
A schematic illustration of the relation between signal variability, functional connectivity and E-I ratio in a computational network model. On whole-brain level, in a model with low E-I ratio (the blue part of the plot), signal variability is high (estimated by PE, in green), and functional connectivity, estimated by wsMI (in orange) and JPE_inv_ (in blue), is low. In a model with E-I balance (shown against a white background), a small change in E-I ratio has a large effect on the outcome measures. As the E-I ratio increases (shifting towards the red part of the plot), signal variability decreases and functional connectivity, measured by both wsMI and JPE_inv_, decreases. Beyond a certain point, a further increase in E-I ratio can lead to a decrease in whole-brain functional connectivity, whereas signal variability remains relatively unchanged. Please note that this plot represents a concept and not actual simulated data

In models with a low E-I ratio, signal variability was high and functional connectivity by wsMI and JPE_inv_ was low. As the E-I ratio increased beyond a certain threshold, signal variability significantly decreased and regions became functionally connected. A schematic representation of the relationships between signal variability, functional connectivity by both wsMI and JPE_inv_ and E-I ratio upon changes in *Vd*_*2*_ is presented in Fig. 8. The transition point was considered representative of a healthy network, balanced between synchrony and asynchrony. Models with higher coupling strength reached this point for lower levels of the E-I determining parameter Vd_2_. After this point, a further increase in E-I ratio on the outcome measures was depended on the filter and time-delay settings.

A healthy neuronal network requires a certain level of variability and flexibility in neuronal activity which can be assessed through measures such as PE, among others (Garrett et al. 2013; Waschke et al. 2021; Yin and Kaiser 2021). Previous studies related neuronal signal variability to behavior (Waschke et al. 2021). Its disruption has been linked to neurological disorders with suspected E-I ratio abnormalities, including dementia (Gaubert et al. 2019; Gomez and Hornero 2010; Hohlefeld et al. 2012; Montez et al. 2009; Shumbayawonda et al. 2020; Sorrentino et al. 2021) and epilepsy (Auno et al. 2021; Meisel et al. 2012). Patients in different stages of the AD continuum, for example, showed decreased signal variability in higher frequency bands and faster time scales (Dauwels et al. 2010; Echegoyen et al. 2020; Maturana-Candelas et al. 2019; Stam 2005), and increased signal variability in the lower frequencies and slower time scales (Gaubert et al. 2019; Maturana-Candelas et al. 2019; Mizuno et al. 2010; Scheijbeler et al. 2022). In epilepsy patients, seizures were characterized by a great reduction in signal variability compared to normal EEG (Bruzzo et al. 2008; Nicolaou and Georgiou 2012). In line with these reports, we showed that lower signal variability (indicating more regular and repetitive activity) was associated with an increased E-I ratio. In contrast to previous studies that found maximal variability at E-I balance in neuronal spiking models (Shew and Plenz 2013; Shew et al. 2009, 2011; Yang et al. 2012), we found the highest level of signal variability at the lowest E-I ratios. The characteristics of the model used for signal variability analyses are, thus, important to consider when determining the relation between signal variability and E-I ratios. To infer changes in signal variability as indicator of altered E-I ratio, it is important to better understand whether healthy human neurophysiological data at whole-brain level is characterized by maximal signal variability, and if so, at what time scale.

The level of signal variability in simulated models with E-I balance varied across regions, despite homogeneous *Vd*_*2*_-values. The distribution of signal variability across the brain was comparable to empirical data, showing low signal variability in posterior and high signal variability in anterior regions (Scheijbeler et al. 2022). The heterogeneity in signal variability is likely explained by the regional differences in the number of connections with other regions (and, therefore, a higher level of excitatory input), as determined by the human DTI-based structural connectivity matrix (Gong et al. 2009). The extent of signal variability change upon a shift in E-I ratio was, in turn, also different across regions. An increase in E-I ratio seemed to decrease the signal variability predominantly in those regions with high intrinsic signal variability, whereas regions with low intrinsic signal variability showed minimal or no changes. In contrast, a decrease in E-I ratio seemed to preferentially decrease signal variability in regions with a high intrinsic level of signal variability. Together, these findings point towards a relationship between E-I ratio and local signal variability on whole brain and regional level.

Higher functional connectivity, measured by both wsMI and JPE_inv_, on whole-brain level related to higher E-I ratios. Maximal functional connectivity was not consistently found for the highest E- I ratios, but was present near a phase transition, at somewhat high E-I ratios, for the extended alpha band oscillations and τ = 50 specifically. A further increase in E-I ratio caused a reduction in functional connectivity. This functional decoupling is possibly explained by the fact that, for these E-I ratios, the neural masses have such high intrinsic activity that it will not be sensitive anymore to input from other regions. Previous studies reported maximal amplitude coupling, phase synchrony and variability in connectivity at E-I balance in a variety of computational network models (Avramiea et al. 2022; Shew et al. 2011; Yang et al. 2012). A deviation from this balance will then, in theory, always result in a reduction of functional connectivity. There are, however, indications from empirical data that healthy brains do not operate exactly at the critical point (characterized by E-I balance), but instead somewhat below that, at a subcritical or quasicritical point (Fosque et al. 2021; Priesemann et al. 2014). This would mean that a relative increase in E-I ratio can increase the functional connectivity until it surpasses the critical point, after which the connectivity will decrease. As for signal variability, it is important to validate this hypothesis in order to use whole-brain average functional connectivity measures as indicators of an abnormal E-I ratio in empirical data.

As suggested here by the hub disruption index results, a change in whole-brain E-I ratio can lead to a region-dependent in- or decrease of functional connectivity. More specifically, we observed a selective disruption of hub-like regions upon a decrease in E-I ratio and a specific increase in functional connectivity by both wsMI and JPE_inv_ in low degree (non-hub) regions as a result of an increased E-I ratio. An increase in E-I ratio could in some scenarios also lead to a decrease in functional connectivity of hub-like regions specifically. The latter functional reorganization has been reported in empirical studies across a number of different diseases with possibly underlying abnormal E-I ratios, including stroke, comatose, and AD patients (Achard et al. 2012; Termenon et al. 2016; Yu et al. 2017). In addition, a dual in- an decrease in functional connectivity have been reported across different studies. For example, EEG and MEG recordings in preclinical and prodromal AD patients have been characterized by increased functional connectivity, in the lower delta and theta band oscillations (Nakamura et al. 2017; Pusil et al. 2019; Ranasinghe et al. 2022a, b), and reduced functional connectivity, usually in higher (alpha, beta, gamma) frequency bands (Briels et al. 2020; Cuesta et al. 2022; Engels et al. 2015; Ranasinghe et al. 2022a, b; Schoonhoven et al. 2022). The differences in connectivity were, compared to controls, also more pronounced in AD patients with than without epileptiform activity (Ranasinghe et al. 2022a, b). As for the whole-brain: after reaching a certain high level of activity, the neuronal populations possibly become insensitive to input from other regions and thereby disrupt the functional connections. Hub-like regions have intrinsically higher activity levels (de Haan et al. 2012) and, therefore, may be the first regions to become functionally decoupled upon an increase in E-I ratio. Whether the combined in- and decrease in functional connectivity as observed in neurological patients represents a whole-brain change in E-I ratio or region-dependent bidirectional changes in E-I ratio remains uncertain. Whereas whole-brain functional connectivity levels already provide information about the underlying E-I balance, analyses of regional functional connectivity changes on different time scales in future studies could provide us with more detailed information about the direction of change in E-I ratio.

To confirm the multi-scale character of JPE_inv_, we correlated JPE_inv_ to both signal variability and functional connectivity by wsMI. We found a strong negative association between signal variability and JPE_inv_ and a strong positive association between wsMI and JPE_inv_. These results indicate that JPE_inv_ indeed captures both signal dynamics and interregional connectivity. For 6–13 Hz, interestingly, the correlation between JPE_inv_ and signal variability and functional connectivity by wsMI was weaker compared to the correlation found for 0.5–70 Hz. It is not so trivial why the strength of the relationship between these measures depended on the filter settings. Signal variability and wsMI exhibited the most distinct/specific relationships with the E-I ratio in this frequency range, mainly driven by the differences in relationship between wsMI and E-I ratio across different time scales. For example, the high functional connectivity by wsMI observed in the alpha band was not detectable in 0.5-70 Hz data. Filtering in the alpha band generally decreased the signal variability, and the resulting increased signal-to-noise ratio possibly increased the sensitivity of wsMI (and JPE_inv_) to capture alpha-band connectivity. Considering that JPEinv combines both measures, it may be expected that JPEinv also shows most distinct relationships to both signal variability and functional connectivity by wsMI for 6–13 Hz.

We hypothesized that the multi-scale character of JPE_inv_ is of potential advantage to identify disrupted E-I ratios from large-scale neurophysiological data (Scheijbeler et al. 2022). The consistently high mean effect sizes for JPE_inv_, in contrast to PE and wsMI, also pointed towards a potential advantage of combining local and interregional signal dynamics in a single metric to measure changes in E-I ratio.

A natural spatial and temporal heterogeneity in E-I ratios likely exists due to intrinsic differences in neuron subtypes, structural wiring, functional importance and dependence on behavioral state (Dehghani et al. 2016; Misic et al. 2010). Previous neuronal population modeling studies have revealed that pathological differences in E-I ratio across brain regions, determined by amyloid- and tau-protein distribution patterns, can explain AD-like neurophysiological signatures, such as spectral slowing (Alexandersen et al. 2023; Ranasinghe et al. 2022; Stefanovski et al. 2019). We here simply applied equal E-I ratio changes across all brain regions, a potential limitation of the study. Due to the interregional differences in number of structural connections, however, we found a similar spatial distribution of signal variability (and functional connectivity by JPE_inv_) in models with E-I balance as observeded in empirical data (Scheijbeler et al. 2022). The regional specificity of E-I ratio changes on the outcome measures could also be explained by the regional difference in connectivity. Overall, these findings suggest that the observed regional signal variability changes and abnormal functional network organization in early stage AD patients, for example, could be explained by abnormal E-I ratio on whole-brain level.

The findings of the study should be considered with the following strengths and limitations in mind. The biophysical nature of the model parameters allowed us to provide meaningful explanations of changes in large-scale dynamics and integrate findings from experimental studies. The realistic model output also allowed us to take a similar analyses approach as other (empirical) studies and therefore facilitates comparison of results. We reported the level of E-I ratio changes that were investigated, which is important for a correct interpretation and translation of findings (Ahmad et al. 2022). We studied the time scale dependency of functional dynamics using multiple approaches and measured simulated brain dynamics on both a regional and whole-brain level. This multi-scale approach provided substantial insight into how E-I ratio changes are reflected in local dynamics, interregional functional connectivity, and functional brain network organization. As any other model, the computational neural mass network model did not capture the full complexity of the brain and, therefore, the translation of the findings to individuals in the clinic remains uncertain. This is especially true for the study of empirical oscillations in other frequencies than the (extended) alpha band. It is important to replicate these findings using alternative models in future studies. Future studies should investigate whether JPE_inv_ is specific to the mechanisms underlying E-I ratio disruption, and if the results of the current study extend to all disorders characterized by changes in E-I ratio such as epilepsy (Houtman et al. 2021; Pani et al. 2021), glioma (Numan et al. 2022), multiple sclerosis (Huiskamp et al. 2022), autism (Bruining et al. 2020; Manyukhina et al. 2022) and schizophrenia (Molina et al. 2020). We also aim to validate the results using empirical data, by comparing JPE_inv_ and related measures between AD patients with and without subclinical epileptiform activity. We used similar outcome measures and symbolic dynamics parameters as in a previous study, and, therefore, volume conduction correction was applied. This is, however, not an issue in ‘model space’ and may have biased our results.

This study proposes a novel nonlinear functional connectivity measure that can help identify E-I ratio changes from short resting-state brain recordings. The metric holds great potential to do so because it captures information about the local dynamics, in terms of variability in symbolic patterns, and the interaction of these dynamics between regions. Previous research has already identified changes in JPE_inv_ in patients at an early stage of AD. Our study extended upon this work by showing that these changes may indeed reflect an abnormal E-I ratio. The identification of a novel E-I measure helps to better understand the mechanisms underlying neurophysiological alterations in AD patients across different stages and, ultimately, provides useful insights for both prognostic and therapeutic purposes.

### Supplementary Information

Below is the link to the electronic supplementary material.

Supplementary file1 (PDF 1615 KB)

## Data Availability

The datasets generated and analyzed during the current study will be publicly available at time of publication at https://github.com/annevannifterick/JPEinv_EI_model. The model is available from and analyses were performed using an in-house developed software (Brainwave, version *0.9.163.26*, available from home.kpn.nl /stam7883/brainwave.html).
