# Supplementary material for: Local signal variability and functional connectivity: Sensitive measures of the excitation-inhibition ratio?
Source: Cogn Neurodyn. 2023 Sep 2;18(2):519–37. doi: 10.1007/s11571-023-10003-x (PMC11061092; doi:10.1007/s11571-023-10003-x)
Supplement: Supplementary file 1 — Supplementary file1 (PDF 1615 KB) [file 11571_2023_10003_MOESM1_ESM.pdf]

Supplementary Information

Local signal variability and functional connectivity: sensitive measures of the excitation-inhibition ratio?

Anne M. van Nifterick[1,2,3,*], Elliz P. Scheijbeler[1,2,3], Alida A. Gouw[1,2,3] Willem de Haan[1,2,3], Cornelis J. Stam[2,3]

1. Alzheimer Center Amsterdam, Neurology, Vrije Universiteit Amsterdam, Amsterdam UMC location VUmc, Amsterdam, The Netherlands
2. Clinical Neurophysiology and MEG Center, Neurology, Vrije Universiteit Amsterdam, Amsterdam UMC location VUmc, Amsterdam, The Netherlands
3. Amsterdam Neuroscience, Neurodegeneration, Amsterdam, The Netherlands

*e-mail-address of corresponding author: a.vannifterick@amsterdamumc.nl

Supplementary Methods

Permutation entropy

Permutation entropy (PE) quantified signal variability by analyzing the distribution of patterns in a time series. For each timepoint *t*, a vector of length *n*: (x_{t+1}, …, x_{t+n}) was assigned to a symbol by ranking the samples within the vector. *L*ength *n* is a predefined number of consecutive data points. Each data point in the vector was assigned a rank, such that the sample with the highest amplitude got rank 1 and the sample with the lowest value rank *n*. A vector with a set of *n* ranks can be considered a unique symbol (or: permutation). After converting the time series in a sequence of symbols, we calculated the probability occurrence of each of these symbols by

$$p(\pi) = \frac{\#\{t|t \leq T-n, (x_{t+1}, \ldots, x_{t+n}) has\ type\ \pi\}}{T-n+1}$$

We calculated the probability of each symbol (or: permutation), which resulted in a probability distribution of *n!* bins. Then, the Shannon information entropy (Shannon, 1948) of this distribution was computed as follows:

$$H(n) = -\sum p(\pi) log\ p(\pi)$$

The maximal entropy value is *log*(*n!*). We normalized the permutation entropy between 0-1 by dividing it by the maximal value: *PE(n) = H(n)/log(n!)*. Simple and repetitive signals have low entropy and more complex, less predictable signals will have high entropy. Whereas Bandt and Pompe showed that the vector length does not strongly influence the PE values, it was recommended to choose *n* in the range between 3-7 and that *n!* is much lower than the total number of time points (Bandt & Pompe, 2002). Therefore, we chose an *n* of 4 in this study.

Weighted symbolic mutual information

Two time series X and Y can share sources of variability which reflect interregional functional connectivity. Here, we measured shared entropy by the weighted symbolic mutual information (wsMI). The symbol-transformed time series were obtained as described for PE. Next, the probably occurrence of each symbol-pair of two brain regions was calculated and used to construct a joint probability matrix with $(n!*n!)^2$ bins. Each identical or opposite symbol-pair (i.e. the diagonals of the

joint probability matrix) was excluded from the further analyses to account for spurious connections in experimental data, as was done previously (King et al., 2013). Then, the joint permutation entropy (JPE) of each combination of two time series was denoted by the Shannon information entropy formula. The weighted symbolic mutual information was defined as follows:

$$wsMI(X;Y) = PE(X) + PE(Y) - JPE(X;Y)$$

Finally, the wsMI was normalized between 0-1 by dividing it by the minimum entropy values of X and Y:

$$wsMI = \frac{wsMI(X;Y)}{\min(PE(X), PE(Y))}$$

A higher wsMI reflects a stronger relationship between time series X and Y.

Inverted joint permutation entropy

The JPE is a multivariate version of signal variability and was used in this study as alternative measure of nonlinear coupling between two brain regions. JPE is a measure of variability in the occurrence of symbol-pairs and was calculated as described above. If two regions are not coupled we will find a greater variability in distribution of symbol-pairs and thus higher JPE values. In contrast, if two regions are coupled, we expect a higher probability occurrence of a certain number of symbol-pairs and thus less variability and lower JPE values. The JPE was also normalized between 0-1 by dividing by *log(n!*n!* – 2*n!* + 1). The -2*n!*+1 number of bins in the probability distribution matrix reflect he diagonals of the probability distribution matrix, which were excluded to address the problem of signal spread in empirical data (as suggested by (King et al., 2013) and applied by (Scheijbeler et al., 2022)). To facilitate an intuitive interpretation of the JPE as a measure of functional connectivity, we inverted the JPE.

$$JPEinv = 1 - JPE$$

If two regions are *weakly* connected, there is a large variability in the distribution of co-occurring symbol probability and this results in higher JPE_{inv} values (close to 1). In contrast, if two regions are *strongly* connected, we expect less variability in co-occurring symbol probabilities and thus lower JPE values (close to 0).

Supplementary Figures

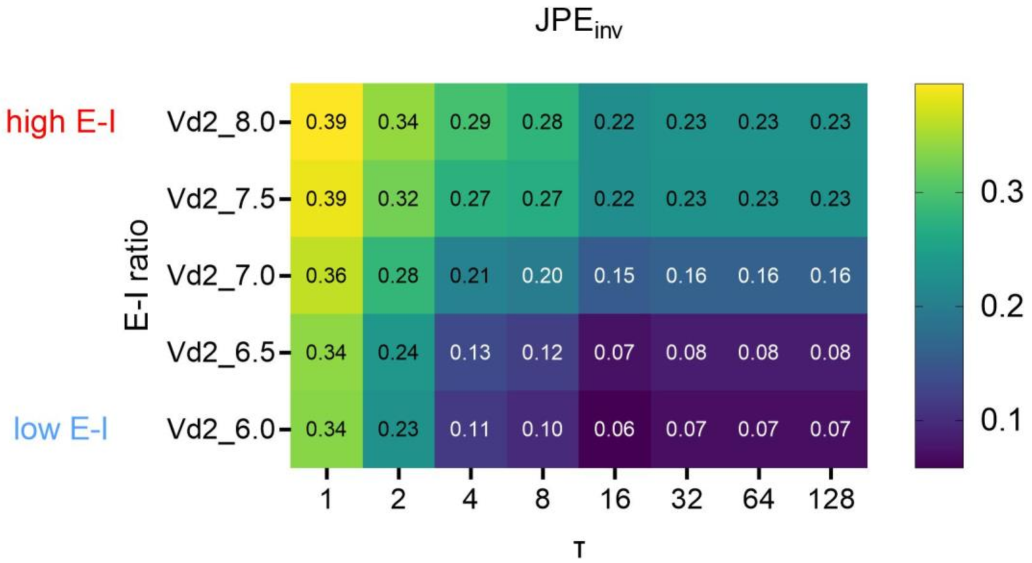

Supplementary Figure 1. Impact of the time delay (τ) parameter on whole-brain average JPE$_{inv}$ is similar for values > 16. Data was generated using an *S*-value of 1.0, dimension (*n*) of 4 and broadband filter (0.5 – 70 Hz). Analyses was performed on 10 model iterations.

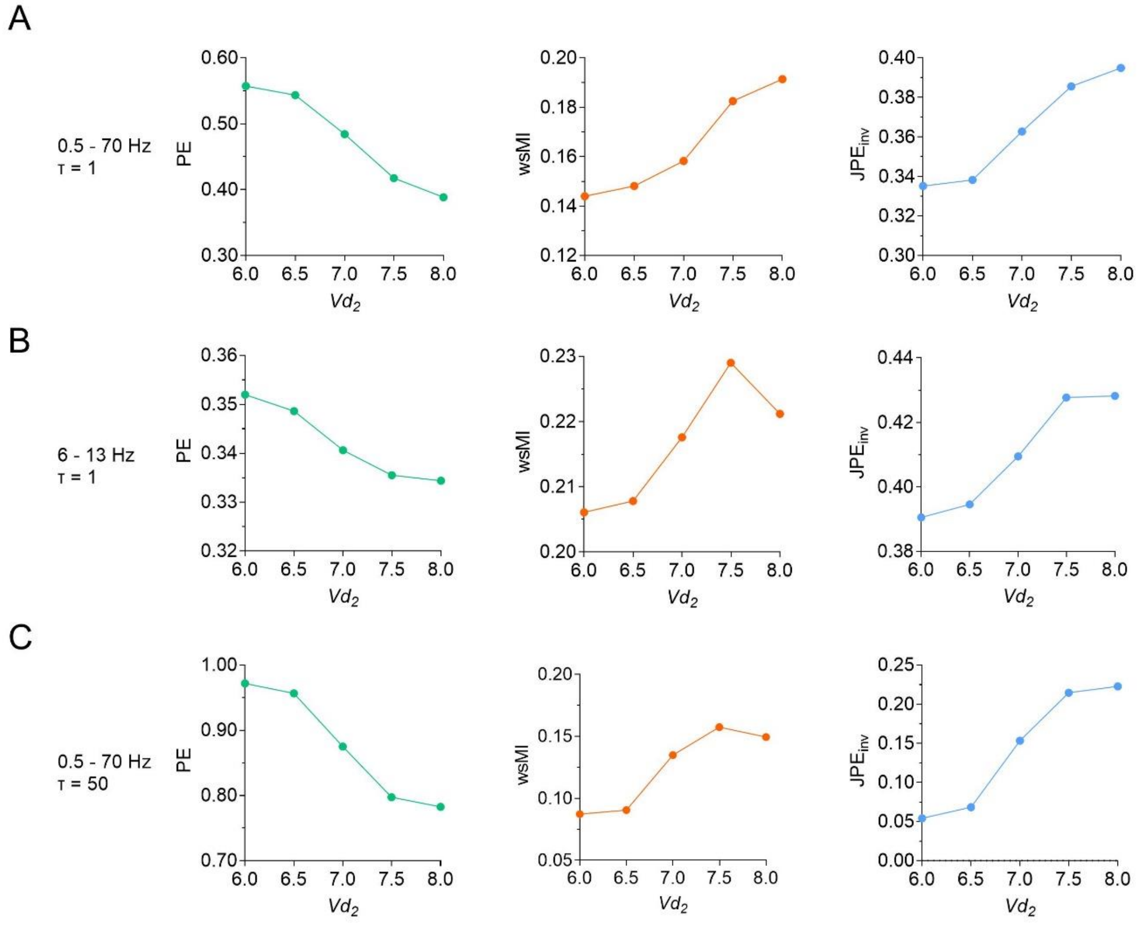

Supplementary Figure 2. PE, wsMI and JPE$_{inv}$ as function of the inhibitory interneuron excitability parameter *Vd₂*. Each data point depicts whole-brain level PE, wsMI and JPE$_{inv}$ results for 5 models across a range of E-I ratios (Vd_2 range: 6.0 – 8.0, with steps of 0.5; and a single coupling (*S*-) value of 1.0). A higher vd2 means a higher E-I ratio. **(A-C)** Each row presents data for different time-scales. A Pearson *r* correlation coefficient analyses (Supplementary Table 2) showed a significant association between the whole-brain average outcome measures PE, wsMI and JPE$_{inv}$ (on the *y*-axis) and model parameter Vd_2 (as indicator of E-I, on the *x*-axis) across all time-scales, except for the relation between wsMI and Vd_2 for 6 – 13 Hz.

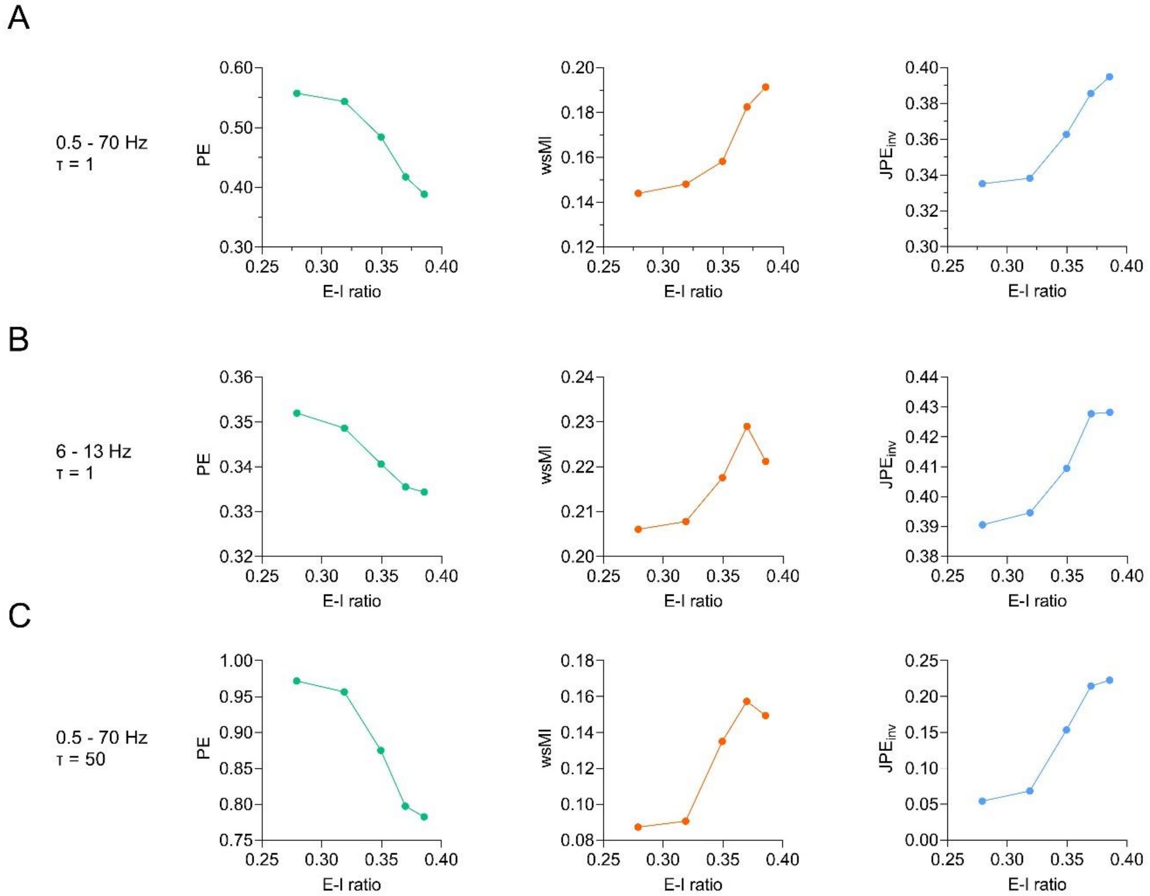

Supplementary Figure 3. PE, wsMI and JPE$_{inv}$ are associated with the E-I ratio. Each data point depicts whole-brain level PE, wsMI and JPE$_{inv}$ results for 5 models across a range of E-I ratios (*Vd$_2$* range: 6.0 – 8.0, with steps of 0.5; and a single coupling (*S*-) value of 1.0). A higher vd2 means a higher E-I ratio. **(A-C)** Each row presents data for different time-scales. A Pearson *r* correlation coefficient analyses (Supplementary Table 3) showed a significant association between the whole-brain average outcome measures PE, wsMI and JPE$_{inv}$ (on the *y*-axis) and the E-I ratio (on the *x*-axis) across all time-scales, except for the relation between wsMI and E-I for 6 – 13 Hz.

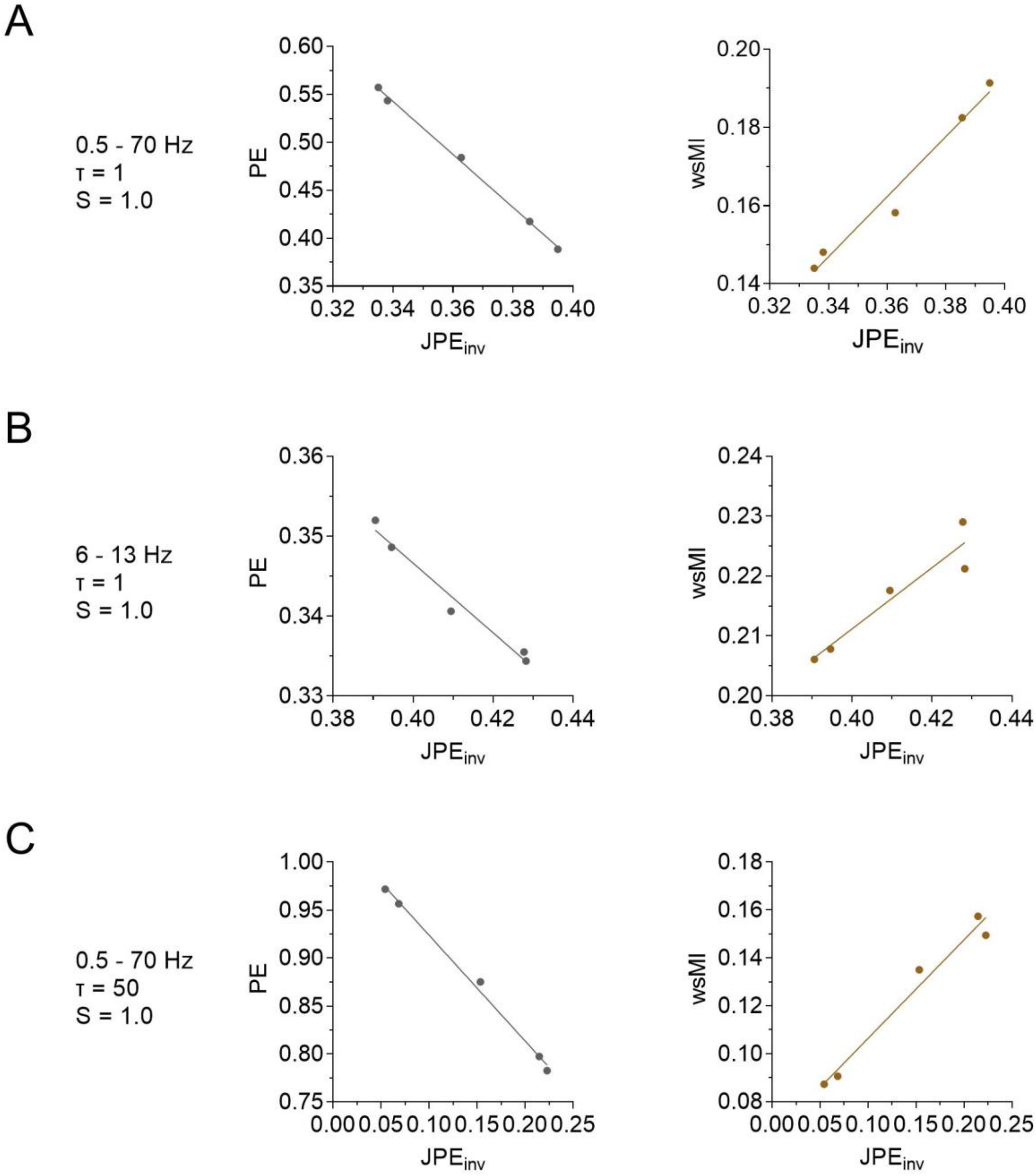

Supplementary Figure 4. PE and wsMI are correlated to $\mathbf{JPE_{inv}}$. Each data point depicts whole-brain level PE, wsMI and JPE_{inv} results for 5 models across a range of E-I ratios (Vd_2 range: 6.0 – 8.0, with steps of 0.5) and a coupling (*S*-) value of 1.0. **(A-C)** Each row presents data for different time-scales. A Pearson correlation analyses (Supplementary Table 4) showed significant associations between whole-brain level PE and wsMI (on the *y*-axis) and JPE_{inv} (on the *x*-axis).

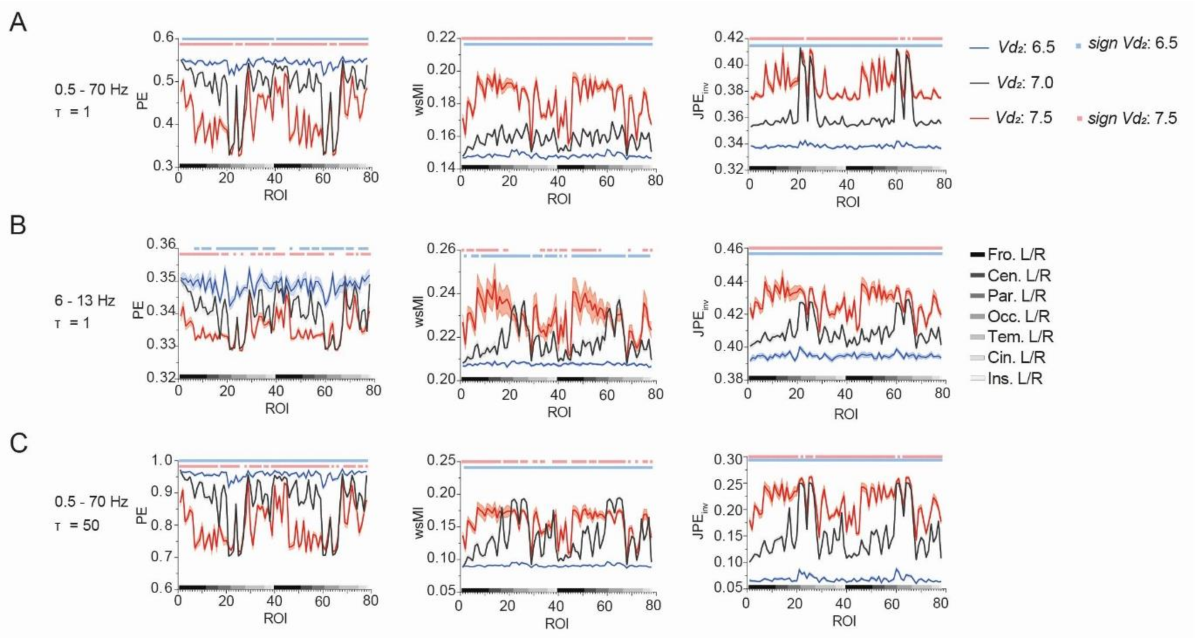

Supplementary figure 5. Regional PE, wsMI and JPE_{inv} for varying levels of E-I. Mean (± 2 * SEM) signal variability (PE), functional connectivity by wsMI and JPE_{inv} per brain region are presented for models with varying levels of E-I (high E-I in red, E-I balance in black, low E-I in blue). Each ROI represents one of the 78 cortical regions in the AAL-atlas (Supplementary Table 1). Multiple Mann-Whitney U tests (FDR corrected) were used to compare outcome measures between high and low E-I ratio models and models with E-I balance per ROI. Analyses were repeated for different time-scales (A-C). Models with high E-I ratios generally showed lower signal variability, higher functional connectivity by wsMI and higher JPE_{inv} compared to a model with E-I balance, although this was dependent on the ROI. Similar but opposite effects were found in general for a model with low E-I ratio compared to a model with E-I balance. PE, permutation entropy; wsMI, weighted symbolic mutual information; JPE_{inv}, inverted joint permutation entropy; E-I, excitation-inhibition; ROI, region of interest; AAL, automatic anatomical labeling; FDR, false discovery rate.

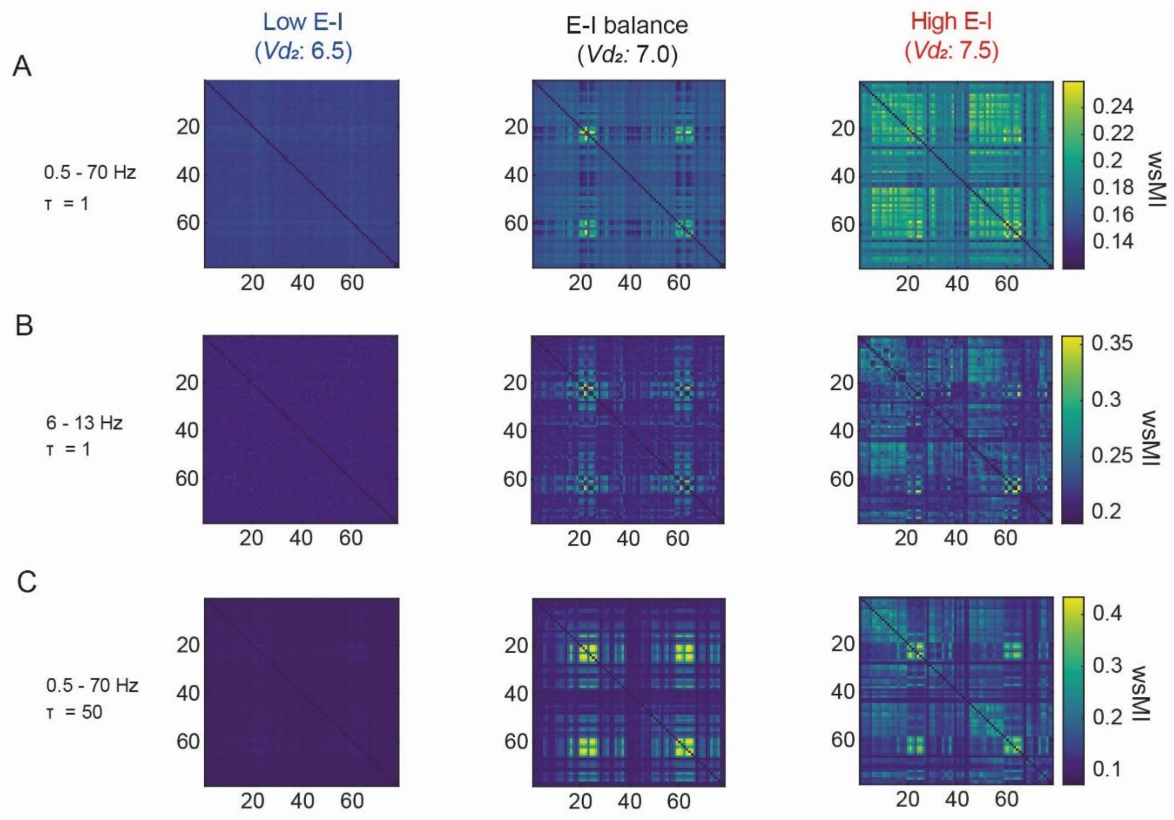

Supplementary figure 6. Pairwise functional connectivity by wsMI for varying E-I ratios. Matrices present the functional connectivity by wsMI between each ROI of the AAL atlas, averaged across 10 iterations. Functional connectivity matrices are visualized for different levels of E-I and across different time-scales (A-C). wsMI, weighted symbolic mutual information; ROI, region of interest; AAL, automatic anatomical labeling;

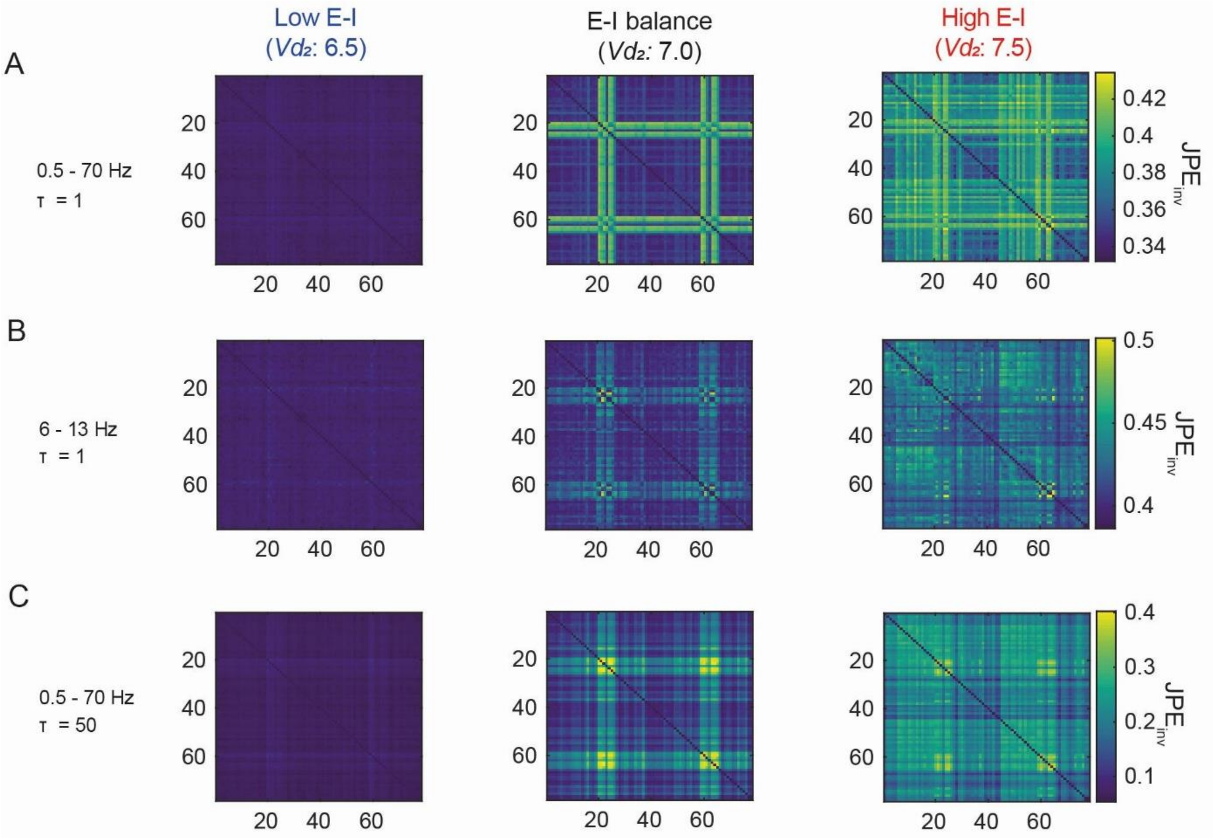

Supplementary figure 7. Pairwise functional connectivity by JPE_{inv} for varying E-I ratios. Matrices present the average connectivity by JPE_{inv} between each ROI of the AAL atlas across 10 iterations. Matrices are shown for different levels of E-I and analyses were repeated for different time-scales (A-C). JPE_{inv}, inverted joint permutation entropy; ROI, region of interest; AAL, automatic anatomical labeling;

Supplementary Tables

Supplementary Table 1. Brain lobes and corresponding region of interest (ROI) number and name according to the AAL atlas (Gong et al., 2009).

Brain lobe	ROI#	Region name left hemisphere	ROI#	Region name right hemisphere
Frontal	1	Rectus_L	40	Rectus_R
	2	Olfactory_L	41	Olfactory_R
	3	Frontal_Sup_Orb_L	42	Frontal_Sup_Orb_R
	4	Frontal_Med_Orb_L	43	Frontal_Med_Orb_R
	5	Frontal_Mid_Orb_L	44	Frontal_Mid_Orb_R
	6	Frontal_Inf_Orb_L	45	Frontal_Inf_Orb_R
	7	Frontal_Sup_L	46	Frontal_Sup_R
	8	Frontal_Mid_L	47	Frontal_Mid_R
	9	Frontal_Inf_Oper_L	48	Frontal_Inf_Oper_R
	10	Frontal_Inf_Tri_L	49	Frontal_Inf_Tri_R
	11	Frontal_Sup_Medial_L	50	Frontal_Sup_Medial_R
Central	12	Supp_Motor_Area_L	51	Supp_Motor_Area_R
	13	Paracentral_Lobule_L	52	Paracentral_Lobule_R
	14	Precentral_L	53	Precentral_R
	15	Rolandic_Oper_L	54	Rolandic_Oper_R
	16	Postcentral_L	55	Postcentral_R
Parietal	17	Parietal_Sup_L	56	Parietal_Sup_R
	18	Parietal_Inf_L	57	Parietal_Inf_R
	19	SupraMarginal_L	58	SupraMarginal_R
	20	Angular_L	59	Angular_R
	21	Precuneus_L	60	Precuneus_R
Occipital	22	Occipital_Sup_L	61	Occipital_Sup_R
	23	Occipital_Mid_L	62	Occipital_Mid_R
	24	Occipital_Inf_L	63	Occipital_Inf_R
	25	Calcarine_L	64	Calcarine_R
	26	Cuneus_L	65	Cuneus_R
	27	Lingual_L	66	Lingual_R
Temporal	28	Fusiform_L	67	Fusiform_R
	29	Heschl_L	68	Heschl_R
	30	Temporal_Sup_L	69	Temporal_Sup_R
	31	Temporal_Mid_L	70	Temporal_Mid_R
	32	Temporal_Inf_L	71	Temporal_Inf_R
	33	Temporal_Pole_Sup_L	72	Temporal_Pole_Sup_R
	34	Temporal_Pole_Mid_L	73	Temporal_Pole_Mid_R
	35	ParaHippocampal_L	74	ParaHippocampal_R
Cingulum	36	Cingulum_Ant_L	75	Cingulum_Ant_R
	37	Cingulum_Mid_L	76	Cingulum_Mid_R
	38	Cingulum_Post_L	77	Cingulum_Post_R
Insula	39	Insula_L	78	Insula_R

Supplementary Table 2. Presented are the whole-brain average *mean ± SD*. Multiple independent t-tests were performed to compare PE, wsMI and JPE_{inv} between a model with low E-I ratio (Vd_2: 6.5, *S*: 1.0) and a model with E-I balance (Vd_2: 7.0), and between a model with high E-I (Vd_2: 8.5, *S*: 1.0) and a model with E-I balance (Vd_2: 7.0, *S*: 1.0). There was a significant difference in the scores for each comparison. Differences, *t*-values, *p*-values and effect sizes no. 1 refer to results of comparisons between a model with low E-I (Vd_2: 6.5) and E-I balance (Vd_2: 7.0), and no. 2 refers to results of comparisons in models with high E-I (Vd_2: 8.5) and models with E-I balance. Cohen's *d* was determined to measure effect sizes and reveals a large effect size for each outcome measure. Compared to PE and wsMI, the mean effect size is higher for JPE_{inv}, especially for 6 – 13 Hz data.

	Low E-I (Vd_2: 6.5)	E-I balance (Vd_2: 7.0)	High E-I (Vd_2: 7.5)	Difference 1 ± *SEM*	Difference2 ± *SEM*	*t(df) 1*	*t(df) 2*	*p*-value 1	*p*-value 2	Effect size 1	Effect size 2	Mean effect size
**0.5 – 70 Hz, τ = 1**												
PE	0.544 ± 0.002	0.484 ± 0.004	0.417 ± 0.006	-0.059 ± 0.001	-0.067 ± 0.002	t(18) = 45.80	t(18)=31.27	< .001	< .001	19.0	13.1	16.1
wsMI	0.148 ± 0.001	0.158 ± 0.001	0.183 ± 0.004	0.010 ± 0.000	0.024 ± 0.001	t(18) = 23.59	t(18) = 19.20	< .001	< .001	10.0	8.6	9.3
JPE_{inv}	0.338 ± 0.001	0.363 ± 0.001	0.386 ± 0.003	0.025 ± 0.000	0.023 ± 0.001	t(18) = 55.25	t(18) = 22.72	< .001	< .001	25.0	10.3	17.7
**6 – 13 Hz, τ = 1**												
PE	0.349 ± 0.001	0.341 ± 0.001	0.336 ± 0.001	-0.008 ± 0.000	-0.005 ± 0.000	t(18) = 29.10	t(18) = 18.42	< .001	< .001	8.0	5.0	6.5
wsMI	0.208 ± 0.000	0.218 ± 0.002	0.229 ± 0.008	0.010 ± 0.001	0.011 ± 0.003	t(18) = 16.97	t(18) = 4.342	< .001	< .001	5.0	1.9	3.5
JPE_{inv}	0.395 ± 0.001	0.410 ± 0.001	0.428 ± 0.005	0.015 ± 0.001	0.018 ± 0.002	t(18) = 28.33	t(18) = 11.68	< .001	< .001	15.0	5.0	10.0
**0.5 – 70 Hz , τ = 50**												
PE	0.957 ± 0.002	0.875 ± 0.004	0.797 ± 0.009	-0.082 ± 0.002	-0.078 ± 0.003	t(18) = 52.62	t(18) = 23.76	< .001	< .001	25.9	11.2	18.6
wsMI	0.091 ± 0.001	0.135 ± 0.004	0.157 ± 0.012	0.044 ± 0.001	0.022 ± 0.004	t(18) = 32.08	t(18) = 5.397	< .001	< .001	15.1	2.5	8.8
JPE_{inv}	0.068 ± 0.002	0.154 ± 0.004	0.214 ± 0.012	0.085 ± 0.002	0.061 ± 0.004	t(18) = 54.07	t(18) = 14.73	< .001	< .001	27.2	6.7	17.0

Supplementary Table 3. The Pearson's *r* correlation coefficients and corresponding *p*-values indicate the strength and significance of the relationship between the whole-brain averaged outcome measures PE, wsMI and JPE_{inv} and the inhibitory interneuron excitability parameter Vd_2. Analyses were performed across a range of Vd_2 values (6.0 – 8.0 in steps of 0.5) and an *S*-value of 1.0.

	**Vd_2 – PE**		**Vd_2 – wsMI**		**Vd_2 – JPE_{inv}**	
	r	*p*	*r*	*p*	*r*	*p*
0.5 – 70 Hz, τ = 1	-.982	.003	.969	.006	.977	.004
6 – 13 Hz, τ = 1	-.975	.005	.853	.066	.966	.008
0.5 – 70 Hz, τ = 50	-.972	.006	.9168	.028	.966	.008

Supplementary Table 4. The Pearson's *r* correlation coefficients and corresponding *p*-values present the strength and significance of the relationship between the whole-brain averaged outcome measures PE, wsMI and JPE_{inv} and the E-I ratio. Analyses were performed across a range of Vd_2 values (6.0 – 8.0 in steps of 0.5) and an *S*-value of 1.0.

	**E-I – PE**		**E-I – wsMI**		**E-I – JPE_{inv}**	
	r	*p*	*r*	*p*	*r*	*p*
0.5 – 70 Hz, $\tau = 1$	-.095	.014	.921	.026	.945	.014
6 – 13 Hz, $\tau = 1$	-.980	.004	.875	.052	.949	.014
0.5 – 70 Hz, $\tau = 50$	-.953	.012	.929	.022	.957	.011

Supplementary Table 5. The Pearson's *r* correlation coefficients and corresponding *p*-values show the strength and significance of the relationship between whole-brain averaged outcome measures PE, wsMI and JPE_{inv}, respectively. Analyses were performed across a range of Vd_2 (6.0 – 8.0 in steps of 0.5) and an *S*-value of 1.0.

	**JPE_{inv} – PE**		**JPE_{inv} – wsMI**	
	r	*p*	*r*	*p*
0.5 – 70 Hz, $\tau = 1$	-.999	< .001	.986	.002
6 – 13 Hz, $\tau = 1$	-.987	.002	.950	.013
0.5 – 70 Hz, $\tau = 50$	-.998	< .001	.986	.002

References

Bandt, C., & Pompe, B. (2002). Permutation entropy: a natural complexity measure for time series. *Phys Rev Lett*, *88*(17), 174102. https://doi.org/10.1103/PhysRevLett.88.174102

Gong, G., He, Y., Concha, L., Lebel, C., Gross, D. W., Evans, A. C., & Beaulieu, C. (2009). Mapping anatomical connectivity patterns of human cerebral cortex using in vivo diffusion tensor imaging tractography. *Cereb Cortex*, *19*(3), 524-536. https://doi.org/10.1093/cercor/bhn102

King, J. R., Sitt, J. D., Faugeras, F., Rohaut, B., El Karoui, I., Cohen, L., Naccache, L., & Dehaene, S. (2013). Information Sharing in the Brain Indexes Consciousness in Noncommunicative Patients. *Current Biology*, *23*(19), 1914-1919. https://doi.org/10.1016/j.cub.2013.07.075

Scheijbeler, E. P., van Nifterick, A. M., Stam, C. J., Hillebrand, A., Gouw, A. A., & de Haan, W. (2022). Network-level permutation entropy of resting-state MEG recordings: A novel biomarker for early-stage Alzheimer's disease? *Network Neuroscience*, 1-19. https://doi.org/10.1162/netn_a_00224

Shannon, C. E. (1948). A Mathematical Theory of Communication. *Bell System Technical Journal*, *27*(4), 623-656. https://doi.org/DOI 10.1002/j.1538-7305.1948.tb00917.x

Zetterberg, L. H., Kristiansson, L., & Mossberg, K. (1978). Performance of a model for a local neuron population. *Biol Cybern*, *31*(1), 15-26. https://doi.org/10.1007/BF00337367
